# Neural network analysis of neutron and X-ray reflectivity data: automated analysis using *mlreflect*, experimental errors and feature engineering

**DOI:** 10.1107/S1600576722002230

**Published:** 2022-04-02

**Authors:** Alessandro Greco, Vladimir Starostin, Evelyn Edel, Valentin Munteanu, Nadine Rußegger, Ingrid Dax, Chen Shen, Florian Bertram, Alexander Hinderhofer, Alexander Gerlach, Frank Schreiber

**Affiliations:** a Institute of Applied Physics, University of Tübingen, Auf der Morgenstelle 10, 72076 Tübingen, Germany; b Deutsches Elektronen-Synchrotron DESY, Notkestraße 85, 22607 Hamburg, Germany

**Keywords:** reflectometry, data analysis, machine learning, Python

## Abstract

The Python package *mlreflect* is demonstrated, which implements an optimized pipeline for the automated analysis of reflectometry data using machine learning. The package combines several training and data treatment techniques discussed in previous publications. The predictions made by the neural network are accurate and robust enough to serve as good starting parameters for an optional subsequent least-mean-squares (LMS) fit of the data. For a large data set of 242 reflectivity curves of various thin films on silicon substrates, the pipeline reliably finds an LMS minimum very close to a fit produced by a human researcher with the application of physical knowledge and carefully chosen boundary conditions. The differences between simulated and experimental data and their implications for the training and performance of neural networks are discussed. The experimental test set is used to determine the optimal noise level during training. The extremely fast prediction times of the neural network are leveraged to compensate for systematic errors by sampling slight variations in the data.

## Abstract 2

A Python-based analysis pipeline for the fast analysis of X-ray and neutron reflectivity data using neural networks is presented.

## 1. Introduction

X-ray and neutron reflectometry (XRR and NR) are established surface scattering techniques that are routinely used to characterize solid and liquid thin films (Tolan, 1999 ▸; Holý *et al.*, 1999 ▸; Braslau *et al.*, 1988 ▸; Russell, 1990 ▸). They offer a non-invasive way of determining the structural, morphological and magnetic properties of a large variety of samples (Neville *et al.*, 2006 ▸; Skoda *et al.*, 2017 ▸; Lehmkühler *et al.*, 2008 ▸) and can also be employed in real time for *in situ* measurements (Kowarik *et al.*, 2006 ▸).

For decades, the conventional way of analyzing reflectivity data has been the iterative least-mean-squares (LMS) or χ^2^ fitting of the data with a theoretical model (Parratt, 1954 ▸; Abelès, 1950 ▸; Heavens, 1955 ▸). However, due to the well known phase problem in scattering, the reconstruction of the scattering length density (SLD) profile from the reflectivity data is inherently ambiguous. This means that this method typically requires significant expertise and prior knowledge about the system, since for all but the simplest cases many possible solutions exist. Even when the solution space is restricted, finding the global minimum is usually very time consuming because there are several local minima on the mean-squared error (MSE) surface. For this reason, various software packages have been developed over the years that use sophisticated minimization algorithms (Björck & Andersson, 2007 ▸; Kienzle *et al.*, 2011 ▸; Nelson, 2006 ▸; Nelson & Prescott, 2019 ▸; Danauskas *et al.*, 2008 ▸; Gerelli, 2016 ▸). However, all of these approaches are iterative in nature and thus usually computationally slow. Recently, machine-learning-based methods have been proposed that could avoid a lengthy search of the MSE surface by providing an immediate guess for the thin-film parameters that is already very close to the ground truth (Greco *et al.*, 2019 ▸; Mironov *et al.*, 2021 ▸; Doucet *et al.*, 2021 ▸; Carmona Loaiza & Raza, 2021 ▸; Greco *et al.*, 2021 ▸) or by encoding the reflectometry data into a latent space where the error surface does not have as many local minima (Andrejevic *et al.*, 2021 ▸).

This paper demonstrates a Python-based reflectivity data analysis pipeline called *mlreflect*, which combines a fully connected neural network regressor with several preprocessing and postprocessing steps for reliably predicting the thickness, roughness and SLD of a thin film layer. The principle of the neural network itself and the preprocessing have been discussed previously (Greco *et al.*, 2019 ▸, 2021 ▸), so here we focus on the differences between simulated and experimental data and show how this knowledge can be used to optimize the obtained results further. We tested the performance of the pipeline on a large experimental data set of 242 XRR curves from different samples by comparing the result of the pipeline with manually supervised LMS fits that include physical knowledge and carefully chosen boundary conditions. This is a quantitative and qualitative difference compared with other similar studies, where most of the performance analysis is done with simulated data. In this context, we discuss the effect that experimental deviations from the theory can have on the training and prediction quality of the neural network. Using an example curve, we show how the extremely fast prediction speed of the neural network can also be leveraged to compensate for small experimental errors.

## 2. Description of the analysis pipeline

**Figure 1:**
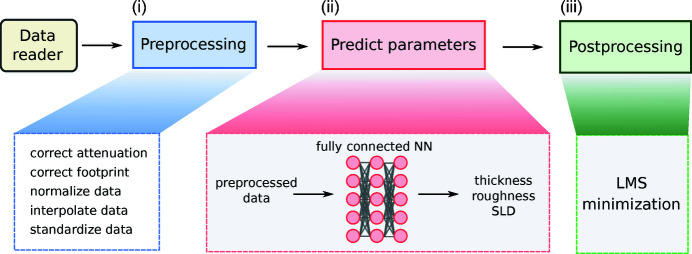
A schematic description of the analysis pipeline. The pipeline consists of three main steps: (i) preprocessing, (ii) parameter prediction via the neural network and (iii) postprocessing. Step (i) includes geometric and other experiment-specific corrections. The data are also normalized, transformed into *q*_*z*_ space, interpolated and standardized. In step (ii), the preprocessed data are fed into a trained fully connected neural network that yields an initial guess for the thin-film parameters. During step (iii), this initial guess is used as starting parameters for a fast Levenberg–Marquardt fit that finds the nearest LMS minimum.

Our proposed analysis pipeline *mlreflect* is written entirely in Python. It is available as open source on GitHub (https://github.com/schreiber-lab/mlreflect) and can also be downloaded directly from the Python Package Index (https://pypi.org/project/mlreflect/). The supporting information contains a step-by-step tutorial in the form of executable *Jupyter* notebooks (and a PDF version thereof). The tutorial, installation instructions and a full API documentation of the *mlreflect* package are hosted online at https://mlreflect.readthedocs.io/en/latest/. The neural network itself is implemented using *TensorFlow* (Abadi *et al.*, 2016 ▸). It uses the matrix formalism implemented in the *refl1d* package (Kienzle *et al.*, 2011 ▸) to simulate the reflectivity data. The workflow of the package can be conceptually separated into three steps: (i) preprocessing, (ii) prediction and (iii) postprocessing, as depicted in Fig. 1 ▸. Each of these steps is described in the following.

During step (i), the reflectivity data are automatically read from their raw format and several types of preprocessing procedures are applied. First, the raw data are converted into the standard *R*(*q*_*z*_) format, where *R* is the normalized reflected intensity and *q*_*z*_ the momentum transfer vector component along the surface normal [momentum transfer *q* = (4π/λ)sinθ, where θ is half the scattering angle and λ is the wavelength of the incident radiation]. The preprocessing operations necessarily depend on how the raw data are saved, but usually the data have to be corrected in some form. In our case, the raw data contain the reflected intensity at different scattering angles, which must be corrected for the varying beam attenuation at different angles. The intensity is then corrected to account for the changing beam footprint on the sample at different angles, which amounts to a multiplication of the data by a geometric factor (Gibaud *et al.*, 1993 ▸). Here we assume a flat sample and a beam with a Gaussian profile but, in principle, corrections for other sample or beam shapes can be implemented at this stage. The data are then normalized by dividing by the highest intensity value and transformed from angular space into *q*_*z*_ space.

After that, the intensity values are interpolated on a log­arithmic scale to 109 equally spaced *q*_*z*_ points ranging from 0.02 to 0.15 Å^−1^, which corresponds to the input size of the neural network. Lastly, each intensity point is standardized individually by subtracting the mean and dividing by the standard deviation of the training set, as described before (Greco *et al.*, 2021 ▸). This ensures that each value of the input vector is on a similar scale. The effect on the general shape of the curves is comparable to multiplying the data by the inverse of the Fresnel reflectivity, 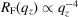 , but, importantly, avoids the divergence for small values of *q*_*z*_, *i.e.* close to and below the total reflection edge (TRE), where the kinematic approximation does not hold (Als-Nielsen & McMorrow, 2011 ▸).

To obtain the initial parameter prediction [step (ii) in Fig. 1 ▸], the preprocessed input vector is fed into the trained neural network model. The neural network is a fully connected model that takes an input of 109 discrete intensity points and outputs three thin-film parameters: the film thickness, the Névot–Croce film roughness (Névot & Croce, 1980 ▸) and the real part of the SLD of the film. The model has three hidden layers with 512 neurons each. The training loss was calculated as the mean-squared error between the normalized predicted and ground truth parameters. This architecture is similar to what has been described in the literature before (Greco *et al.*, 2019 ▸, 2021 ▸; Doucet *et al.*, 2021 ▸), but to reduce the training and inference times the number of parameters was reduced. The model was trained with 250 000 simulated reflectivity curves with a batch size of 512. For every batch, uniform noise and curve scaling were applied to each curve to avoid overfitting, as described previously (Greco *et al.*, 2021 ▸). The optimal noise level during training was identified to be 0.3, which will be discussed in more detail later. Finally, the inputs were standardized as described above.

The training data were generated assuming a sample structure consisting of a thin film on top of an oxide-capped silicon substrate, with air as the ambient medium and X-rays as the probe. The thin-film parameters in the training data spanned a large range of 20–1000 Å for the thickness, 0–100 Å for the roughness and 1–14 × 10^−6^ Å^−2^ for the SLD. We restricted the roughness to values no higher than half the thickness since scenarios with a high relative roughness are not well described by the theoretical model used. A similar approach could easily be employed for neutrons or other sample structures by retraining the neural network with different training data. We also expect this approach to work for a larger number of layers, as long as the trained parameter space does not create too many ambiguous solutions, *i.e.* the number and range of fitting parameters should remain similar. For a larger parameter space, a larger *q*_*z*_ range might be necessary to reduce ambiguity in the data. In our case, the *q*_*z*_ range was limited to avoid conflicts with the Bragg peaks of organic molecules around 0.3 Å^−1^ which are not described by the slab model.

Lastly, during step (iii), the initially predicted thin-film parameters are fed into an LMS minimizer to obtain the parameters that produce the best fit. Since the initial predictions are already very close to the ground truth, we chose a simple Levenberg–Marquardt minimizer (Moré, 1977 ▸) over a more powerful, but slower, algorithm.

## 3. Performance test on thin films

**Figure 2:**
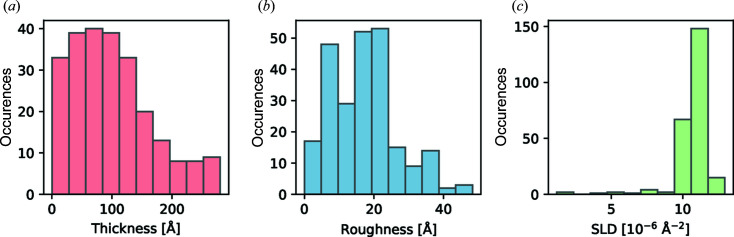
Ground truth distribution of the three sample parameters, (*a*) thickness, (*b*) roughness and (*c*) SLD, within the experimental test set of 242 XRR curves. The parameters were obtained by a conventional LMS fit.

The performance of the analysis pipeline was tested on 242 experimental XRR curves from *in situ* and *ex situ* experiments on nine organic thin films on Si/SiO_*x*_ (1–79 curves per sample at different thicknesses). The distributions of thickness, roughness and SLD of the films within this test set are shown in Fig. 2 ▸. The measurements were conducted using three different synchrotron radiation sources, namely the European Synchrotron Radiation Facility (ESRF; Smilgies *et al.*, 2005 ▸), DESY (Seeck *et al.*, 2011 ▸) and the Swiss Light Source (SLS; Patterson *et al.*, 2005 ▸), as well as using our own laboratory source. To obtain a benchmark, each reflectivity curve was first fitted on a logarithmic scale with an LMS fit based on the commonly used differential evolution algorithm (Storn & Price, 1997 ▸) and manually chosen initial values and bounds for each parameter. The thin-film model used for the fit was the same as that used for training the neural network. In the following analysis, we assume that these manually fitted parameters represent the ‘ground truth’, and thus the performance of our pipeline will be measured as the absolute error with respect to this ground truth.

In the following, we compare the ground truth with the prediction results of the neural network, as well as with the results of a subsequent automated LMS fit using the predicted parameters. Across all 242 curves, the neural network predictions have a median absolute error (median percentage error) of 6.0 Å (7.1%) for the film thickness, 2.0 Å (12.4%) for the interface roughness and 0.72 × 10^−6^ Å^−2^ (6.8%) for the SLD. This is a significant improvement on our first published model (Greco *et al.*, 2019 ▸), both on an absolute scale and on a relative scale, since the possible ranges for the thickness and roughness parameters have been greatly expanded. Thus, the network is generalized over a larger parameter space compared with previously published results. We note that, since all of our data stem from organic thin films, the SLDs in the test set are mainly clustered around 10–13 × 10^−6^ Å^−2^. Nevertheless, we assume that our results are not specific to the SLD range of the test data, since the network was trained equally with SLDs in the range 1–14 × 10^−6^ Å^−2^. We also highlight the fact that the data set contains curves with a high roughness-to-thickness ratio where the Kiessig oscillations are strongly damped. Among the emerging solutions offered in this field, discussions about the performance on curves with few to no features are mostly absent. This is of course due to the challenge of extracting information from data that inherently contain less information. Nevertheless, the network presented here also performs well on experimental data with high relative roughness.

**Figure 3:**
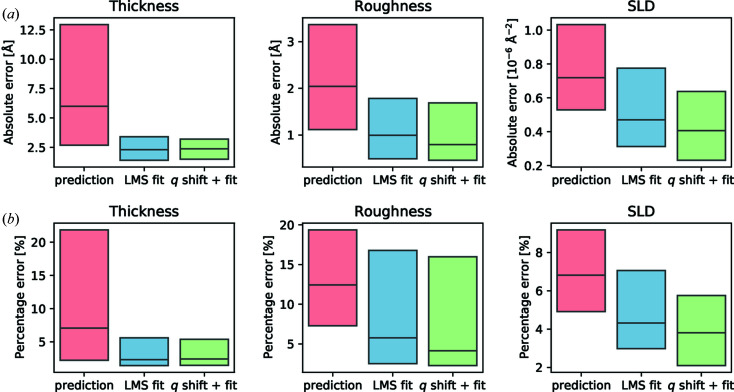
(*a*) Box plots of the absolute errors for 242 measured reflectivity curves for each of the three predicted parameters. The upper and lower edges of the boxes represent the first and third quartiles, respectively, with the horizontal line inside the boxes denoting the median. The blue boxes represent the error compared with the pure neural network predictions. The pink boxes represent the error after applying a simple LMS minimization using the neural network predictions as starting parameters. The green boxes show the error for the case when a *q*_*z*_ shift optimization has been performed before the LMS fit. (*b*) The same box plots of the median error but this time as a percentage of the ground truth. All results were obtained for a training noise level of *n* = 0.3.

The next step in the pipeline is to refine these results further via an LMS fit, using the predictions from the neural network as starting parameters. Since the predictions are robust and already quite close to the ground truth there is no need for powerful but slow minimization algorithms such as genetic or differential evolution algorithms, which are normally employed to find the global minimum. Thus, finding the minimum takes only a fraction of a second per curve and can be fully automated. After this refinement procedure, the median absolute error (median percentage error) was even closer to the ground truth, at 2.3 Å (2.3%) for the thickness, 1.0 Å (5.8%) for the roughness and 0.47 × 10^−6^ Å^−2^ (4.3%) for the SLD. A comparison of the error distributions before and after refinement is shown in Fig. 3 ▸. A detailed breakdown of the prediction error with respect to each parameter can be found in Figs. S2–S10 in the supporting information.

The residual error can be attributed to the fact that every fit has a finite accuracy and hence the ground truth itself contains a certain error. We roughly estimate this error to be at least ±10% for each parameter, which would be comparable to the reported error of the neural network. Thus, these results show that the analysis pipeline as described above performs similarly to a human researcher in most circumstances. However, the results were obtained much faster than via a human-guided fit. Excluding the time it took to train the neural network (about 20 min for a given sample structure), the initial parameter predictions of the 242 curves were obtained after only 1 s, with about two additional minutes for the further refinement steps, resulting in a total fitting time of about 0.4 s per curve. In contrast, producing the ground truth fits took about 6 h because of the need to select fitting boundaries carefully to prevent the fit from running into non-physical minima.

## 4. Differences between simulated and experimental data

A well known property of artificial neural networks is that they require large amounts of representative training data to learn a generalized model and not overfit the training set. In the context of the work presented here, *i.e.* supervised learning using scattering data, this would mean acquiring thousands of scattering patterns from a large variety of different samples and analyzing them manually to create the training set. Since this is quite a time-consuming and challenging task, neural network models in the field of scattering physics are typically trained with simulated data based on well established theoretical models. In most cases, the simulation is additionally modified with certain artifacts, such as noise, to mimic experimental conditions better. However, to what degree this is necessary is difficult to estimate since the only available metric is typically the performance on other simulated data (validation loss), which is expected to decrease with increasing perturbations.

In this study, we investigated how applying uniform noise to the training data affects the neural network performance on our large experimental data set of 242 curves. We trained 11 copies of the same neural network (as described above) with training data with different noise levels *n*, where each data point 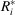 in the noisy curve was sampled uniformly between the values *R*_*i*_(1 − *n*) and *R*_*i*_(1 + *n*). Thus, *n* denotes the maximum relative change in a given data point *R*_*i*_ of a given simulated curve. The value of *n* for each trained model ranged from 0 to 0.5 in increments of 0.05. The applied uniform noise is not meant to model a specific physical noise type, such as Poisson noise for counting statistics. Rather, uniform noise was chosen as a *q*-independent catch-all noise that affects the whole curve equally and thus makes the neural network robust against errors across the entire *q* range.

**Figure 4:**
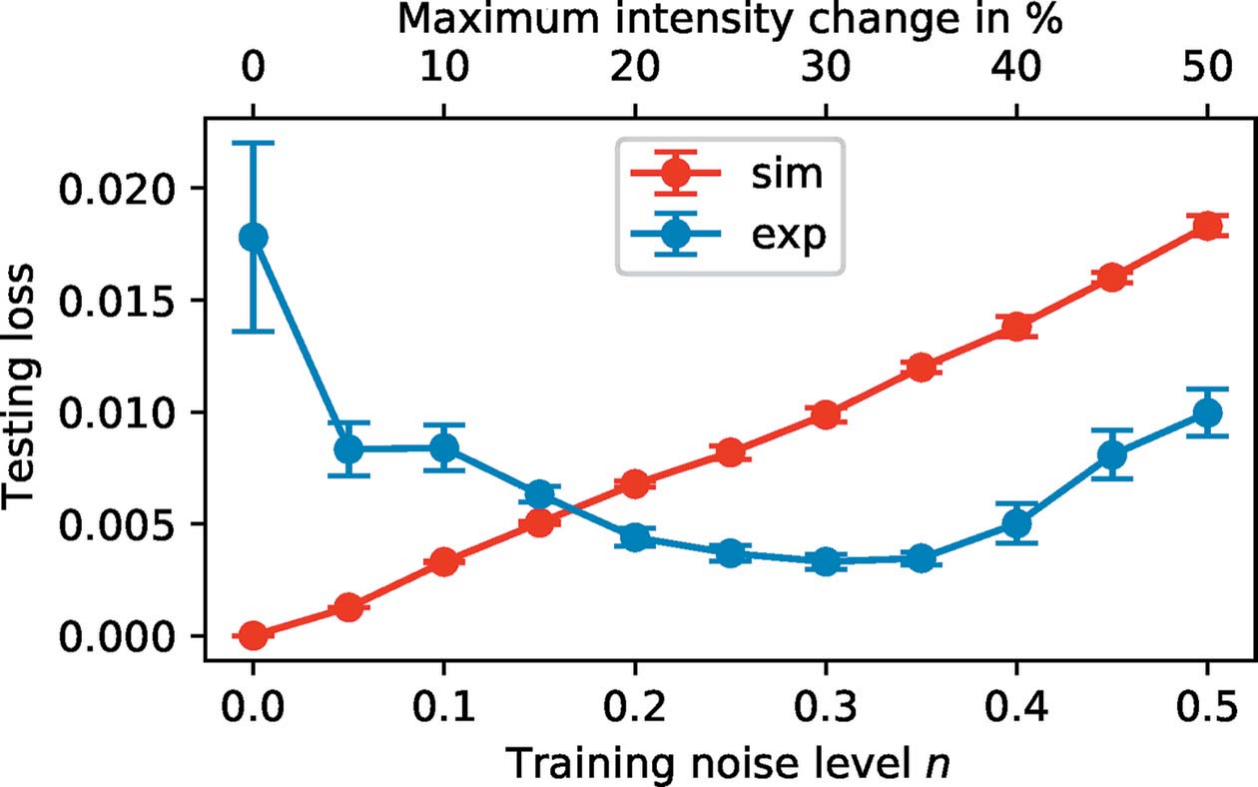
A comparison of the testing loss calculated from a simulated test set (100 000 curves, red) and an experimental test set (242 curves, blue) for different levels of uniform noise *n* that were applied to the training data. For each noise level a separate model was trained. With increasing noise level, the loss from the simulated data increases linearly, while the loss from the experimental data shows a clear minimum at noise levels of 0.3–0.35. The error bars represent the standard deviation from five independent training repetitions.

Fig. 4 ▸ shows a comparison of the losses calculated with a simulated test set and with the experimental test set for each model. Since the loss is calculated as the mean-squared error of all three (normalized) sample parameters, it is a unitless measure for the accuracy of the model. For *n* = 0, the simulated test set shows a loss close to zero (∼10^−7^), whereas the loss based on the experimental data is about five orders of magnitude higher. This shows that, without any noise, the neural network significantly overfits the simulation and thus performs suboptimally on real data. As expected, the loss of the simulated data increases monotonically with increasing noise. However, the performance on the real data improves significantly with increasing noise up to a noise level of 0.3–0.35. Beyond this, still higher noise levels seem again to worsen the performance. This very clearly demonstrates that there exists an optimal noise level for which the added noise acts as an effective regularization technique that prevents overfitting. If the noise level is too high, however, the consequent lack of information is likely to be detrimental to the training. Thus, we identified *n* = 0.3 to be the ideal noise level for data similar to our testing set, which notably contains data from different X-ray sources. Fig. S1 in the supporting information shows that the optimal training noise does not change significantly for subsets with different noise levels (0.1–0.5) within the experimental test set. Thus, we set the default value of the noise level in our analysis pipeline to 0.3. Data sets that differ significantly from our test set in terms of experimental artifacts might of course produce slightly different results, although we expect the general trend to be the same. This highlights the importance of having a large experimental test set with representative experimental artifacts, since metrics based only on simulated data are clearly not sufficient to evaluate the training progress.

## 5. Influence of systematic measurement errors

All reflectometry measurements are performed with a finite accuracy due to various error sources. These errors are detrimental to the experiment and can impede the extraction of information from the data, and therefore should be avoided or minimized as much as possible. However, a finite error inevitably remains for every measurement. Among the possible statistical errors are Poisson noise from counting statistics, the angular resolution of the diffractometer and the spectral resolution of the source. Among the systematic errors are, for example, the convolution of the data with the slit functions, the accuracy of the sample alignment and the accuracy of the footprint correction (*i.e.* how accurately the beam and sample shape can be determined in practice).

**Figure 5:**
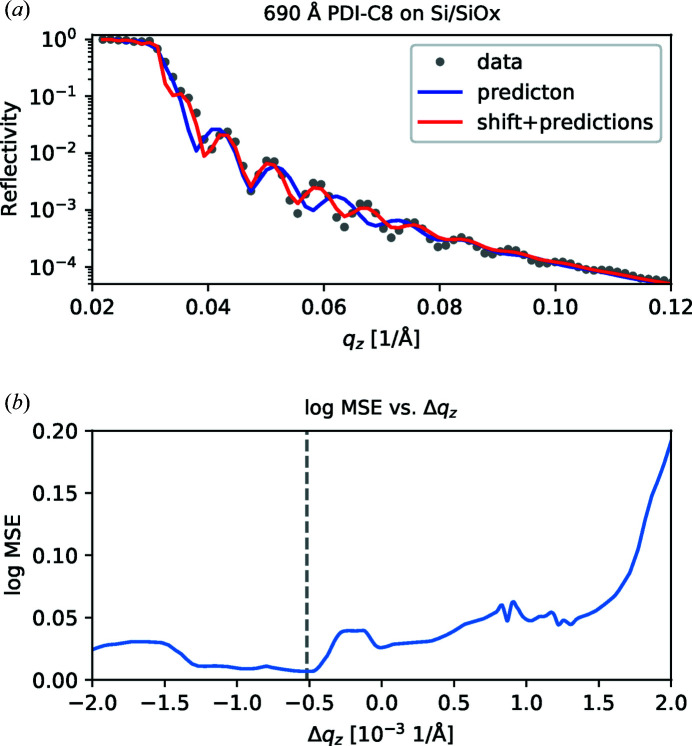
(*a*) A comparison of the neural network predictions from reflectivity data from a 690 Å thick PDI-C8 film on Si/SiO_*x*_. The blue curve shows the native prediction and the red curve shows the prediction after the data were shifted by Δ*q*_min_ = 5.2 × 10^−4^ Å^−1^ before the interpolation step. It is apparent that the latter is in much better agreement with the data. (*b*) The log MSE between the predicted curve and the data for different Δ*q*_*z*_. The minimum MSE at Δ*q*_min_ is indicated by the dashed line.

Having imperfect data obviously has an impact on the analysis, since the data deviate from the ideal physical model they are compared with. Since the neural network model presented here is trained to solve a very particular task that assumes well defined data, these errors can negatively impact the prediction quality. In general, it is easier to make the neural network robust against statistical errors by introducing them during training, as described before. However, sometimes systematic errors, such as a small misalignment, can also seriously misguide the machine learning prediction, as shown in Fig. 5 ▸. Therefore, it would be useful to correct or compensate for some of these errors during inference time after the data have been acquired.

As a solution, we propose an automated method for sampling through slight variations in the data, exploiting both the sensitivity and speed of our neural network model. Since the neural network assumes data that conform to an idealized physical model, it might fail if the data contain anomalies with respect to that model. Since predictions with the neural network are very fast, it is possible to scan through thousands of modified reflectivity curves in less than a second. For each of these variants, the log MSE between the data and the predicted curve can be calculated and only the one with the lowest error is subsequently selected. We demonstrate an implementation of this method that identifies small systematic alignment errors and automatically applies an appropriate shift to the data.

Fig. 5 ▸(*a*) shows an XRR measurement of a 690 Å thick film of *N*,*N*′-dioctyl-3,4,9,10-perylenedicarboximide (PDI-C8) on Si/SiO_*x*_, which was measured and tested in addition to the 242 test curves. Here, in contrast to the previously shown test set, the normal pipeline as described above did not converge to the correct minimum. The reason for this is the much higher thickness of the film, which leads to denser Kiessig oscillations in the data. This, in turn, creates many narrow minima on the MSE surface for the LMS algorithm to get trapped in. As a result, the neural network prediction needs to be even closer to the ground truth for the subsequent fit to converge. Table 1 ▸ shows the predicted thin-film parameters in comparison with the ground truth. A possible reason for the suboptimal neural network prediction might be small imperfections in the data due to finite measurement errors, such as a small variation in sample alignment. In regions of high derivatives, even a small shift in the data along the *q*_*z*_ axis can lead to strong differences in the observed intensities at a given *q*_*z*_ value, even on a logarithmic scale. Of course, if the data have dense oscillations, this effect becomes more pronounced. For models trained on simulated data this can be critical, since normally a substantial change in certain input neurons, especially near the TRE, corresponds to important information and will be interpreted by the network accordingly. To check whether this can be remedied, we shifted the *q*_*z*_ values during the interpolation step by a small value Δ*q*_*z*_ and repeated the prediction. This was done 1000 times with randomly sampled Δ*q*_*z*_ ranging from −1 × 10^−3^ to 1 × 10^−3^ Å^−1^. Then, for each prediction, the quality of the prediction was evaluated by calculating the log MSE between the corresponding simulation and the measured curve.

When plotting the log MSE between the prediction and the input against Δ*q*_*z*_ (Fig. 5 ▸), we observed a value Δ*q*_min_ = 5.2 × 10^−4^ Å^−1^ for which the log MSE shows a clear minimum. From Fig. 5 ▸(*a*) it is apparent that the predicted curve based on the shifted data shows much better agreement with the data than the normal prediction. The corresponding predicted parameters for Δ*q*_min_ (shown in Table 1 ▸) are much closer to the ground truth values (comparable to the values given in the previous section). This indicates that there exists a certain shift Δ*q*_min_ that can (at least partially) compensate for the experimental error. This is especially valuable since it allows the pipeline to continue with the LMS refinement step, which ultimately leads to a near-perfect fit.

Note that Δ*q*_min_ is very small, corresponding to a change in the angle of incidence of only about 4 × 10^−3^° for a wavelength of 1.54 Å. It seems intuitive that such a small shift in the data could be caused by a variety of the above-mentioned error sources. However, although Δ*q*_min_ is seemingly small, because of the high derivatives close to the TRE and the Kiessig fringes, shifting the data by Δ*q*_min_ still has a noticeable effect on each data point. For conventional LMS fitting this might not seem critical at first, since the MSE surface probably has a minimum close to the real one in terms of the film thickness. However, for the roughness and density parameters this might not be the case, and thus most fitting programs allow the user to shift the data manually if necessary.

While in principle any type of modification like this could conceivably be applied to the data to scan for the lowest MSE, we observed significantly better results with this method rather than, for example, adding Gaussian noise. This is because a translation of the curve preserves most of the information in the data while still varying every data point, in contrast to Gaussian sampling which is *q*-independent and inevitably destroys information.

To test the stability of this method, we applied the Δ*q*_*z*_ sampling procedure to all 242 curves discussed in the previous section (where the pipeline already succeeded) and compared the results with the original mean absolute error. When looking at Fig. 3 ▸, it becomes clear that scanning for Δ*q*_min_ did not harm the mean absolute error, but instead even improved the results slightly for all three parameters. While the log MSE of the predictions is already very close to the minimum, most of the data probably still have a finite alignment error, but this was not sufficient to affect the prediction. Hence, this could still be compensated for by applying a small shift, ultimately leading to an even better fit. Because this screening for Δ*q*_*z*_ yielded significant improvements on some data and was relatively fast, we decided to add this routinely to the analysis pipeline.

## 6. Fourier transforms as a method for feature engineering

The specular reflectivity from a single layer on a substrate well above the critical angle can be approximately described by 

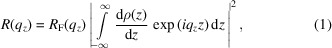


*i.e.* the product of the Fresnel reflectivity from a flat surface and the squared Fourier transform of the SLD contrast of the sample along the surface normal (Als-Nielsen & McMorrow, 2011 ▸; Sivia, 2011 ▸). Although the phase of the Fourier transform is lost by taking its absolute square, the inverse Fourier transform of *R*(*q*_*z*_)/*R*_F_(*q*_*z*_) still carries some important information, such as the frequency of the Kiessig oscillations (and thus the film thickness). As a result, performing an inverse Fourier transform on the reflectivity data presents itself as an obvious way of creating additional input features that may facilitate the neural network training.

To test this hypothesis, we trained a neural network model with an additional preprocessing step before the first layer that performs a fast Fourier transform on the standardized input and adds the real and imaginary Fourier components to it, leading to a input layer size of 219 neurons. All other model parameters and training ranges were kept the same as described above. When testing the trained model on the 242 experimental curves, we found that the model performed similarly to the model without the added Fourier transform. The median absolute errors (median percentage errors) were 6.2 Å (8.9%) for the film thickness, 2.3 Å (13.3%) for the interface roughness and 0.76 × 10^−6^ Å^−2^ (7.2%) for the SLD, which are 4, 19 and 6% higher, respectively, than for the base model.

From this we conclude that the base model (without the added Fourier transform) had probably already learned to extract all available frequency information implicitly from the data, and adding the Fourier components explicitly does not lead to a better training result. The reason why the results are slightly worse when the Fourier transform is added might be the increased number of trainable parameters due to the larger number of neurons in the model. Thus, more parameters need to be optimized to achieve the best training result, which is generally a more difficult task. For these reasons, and the added computational requirements during both training and inference time, we decided not to include the Fourier transform layer in the default neural network layer of our analysis pipeline. Nevertheless, we do not rule out that a suitable implementation of the Fourier transform could be beneficial for certain scattering geometries.

## 7. Conclusion

We have demonstrated an optimized analysis pipeline, *mlreflect*, based on machine learning for the automated analysis of reflectivity data. We have tested our pipeline on a large data set of 242 XRR curves, containing *in situ* and *ex situ* measurements of organic thin films on Si/SiO_*x*_ substrates, where it showed a performance comparable to a manually supervised least-mean-squares fit for most of the data. Therefore, we conclude that *mlreflect* is a useful tool for the automated pre-screening or even on-the-fly analysis of reflectivity data.

We have also discussed that, for the effective evaluation of trained machine learning models, a sufficiently large experimental data set is necessary. Most studies so far have mainly focused on the performance of the model with regard to simulated data and include only a few, if any, experimental test data. However, this may be misleading, since our results clearly show that the performance on simulated data cannot easily be generalized to experimental conditions.

We have shown the influence of possible systematic errors (such as misalignment) on the reflectivity data and how the prediction speed of the neural network model can be exploited to improve the overall performance by transforming the data slightly. Our results highlight the necessity of accounting for these differences between simulated theoretical models and real data in order to obtain stable results.

Although the results shown here were demonstrated with systems of one layer on an Si/SiO_*x*_ substrate, the neural network model could easily be retrained to determine any single layer of any sample structure. While determining multiple layers at once is possible in principle and has been demonstrated before, this type of neural network architecture might not be ideal to tackle this type of inverse problem with multiple solutions, since they map exactly one solution to a given input. Therefore, architectures that yield probabilities as an output might be more suitable for multi-layer problems.

## Supplementary Material

Supporting information including additional figures. DOI: 10.1107/S1600576722002230/vh5156sup1.pdf

Tutorial files for the mlreflect package. Includes commented demo code in the form of Jupyter notebooks and PDF transcripts. DOI: 10.1107/S1600576722002230/vh5156sup2.zip

## Figures and Tables

**Table 1:** Predicted and fitted thin-film parameters based on the reflectivity data of a PDI-C8 film on Si/SiO_*x*_ (shown in Fig. 5 ▸) The ground truth labels were obtained via a manually supervised LMS fit. After applying the described *q*_*z*_ variation, the prediction results improved significantly. A subsequent LMS refinement only led to comparatively small improvements.

	Thickness (Å)	Roughness (Å)	SLD (× 10^−6^ Å^−2^)
Ground truth	688.3	27.1	10.5
Prediction	536.7	30.3	11.2
Shift + prediction	690.8	31.0	11.0
Shift + prediction + fit	690.5	27.5	10.8
