# Supplementary material for: Neural network analysis of neutron and X-ray reflectivity data: automated analysis using mlreflect, experimental errors and feature engineering
Source: J Appl Crystallogr. 2022 Apr 2;55(Pt 2):362–9. doi: 10.1107/S1600576722002230 (PMC8985606; doi:10.1107/S1600576722002230)
Supplement: Supplementary file 1 [file j-55-00362-sup1.pdf]

JOURNAL OF APPLIED CRYSTALLOGRAPHY

Volume 55 (2022)

Supporting information for article:

Neural network analysis of neutron and X-ray reflectivity data: automated analysis using *mlreflect*, experimental errors and feature engineering

Alessandro Greco, Vladimir Starostin, Evelyn Edel, Valentin Munteanu, Nadine Rußegger, Ingrid Dax, Chen Shen, Florian Bertram, Alexander Hinderhofer, Alexander Gerlach and Frank Schreiber

Neural network analysis of neutron and X-ray reflectivity data: automated analysis using *mlreflect*, experimental errors and feature engineering

# – Supporting Information –

Alessandro Greco[a], Vladimir Starostin[a], Evelyn Edel[a], Valentin Munteanu[a], Nadine Rußegger[a], Ingrid Dax[a], Chen Shen[b], Florian Bertram[b], Alexander Hinderhofer[a], Alexander Gerlach[a], and Frank Schreiber[a]

[a] Institute of Applied Physics, Auf der Morgenstelle 10, University of Tübingen, 72076 Tübingen, Germany

[b] Deutsches Elektronen-Synchrotron DESY, Notkestr. 85, 22607 Hamburg, Germany

I. OPTIMAL TRAINING NOISE LEVELS

Figure 4 of the main manuscript shows the loss on the experimental dataset of 242 curves for 11 different neural network models where the training data was modified with different amounts of uniform noise. The results show that there seems to be an optimal noise value of about 0.3 where the loss for the experimental data has a minimum.

An interesting question arises about how this value is related to the amount of noise in the experimental data. To investigate this, the test data was separated into four groups with varying amounts of noise. While the noise in the data is not uniformly distributed, an equivalent noise level (ENL) can be calculated by subtracting the ground truth fit from the data and taking the absolute mean of all data points. Figure S1a shows the distribution of the ENL across the entire dataset and how the distribution was split into the four subsets with a different ENL. Figure S1b shows the optimal training noise (for which the loss had a minimum) for each of the four categories as well as the entire dataset. The error bars represent the standard deviation of five independent training repetitions. Evidently, the ENL of the data does not seem to have a strong influence on the optimal noise level except for the 0.4–0.5 category, where it is slightly lower. This is due to the main source of error in the data not being statistical (e.g. Poisson noise), but rather systematic in nature (e.g. the fit does not fully describe the data). Since the role of the uniform noise on the training data is not to mimic the noise in the data, but to account for these systematic deviations, the entire dataset benefits from a similar training noise level.

However, for data with significantly higher statistical noise than our dataset, it could be possible that optimal training noise is different.

II. DETAILED PREDICTION ERROR HISTOGRAMS

This section shows detailed histograms of the absolute error distribution of each parameter with respect to the ground truth (GT) of a given parameter, which expands on the condensed form shown in Figure 3 of the main manuscript. Here, only the results of the full pipeline are shown (neural network + q shift + LMS fit). Figure S2–S4 show the errors with respect to the GT thickness, Figure S5–S7 with respect to the GT roughness and Figure S8–S10 with respect to the GT SLD.

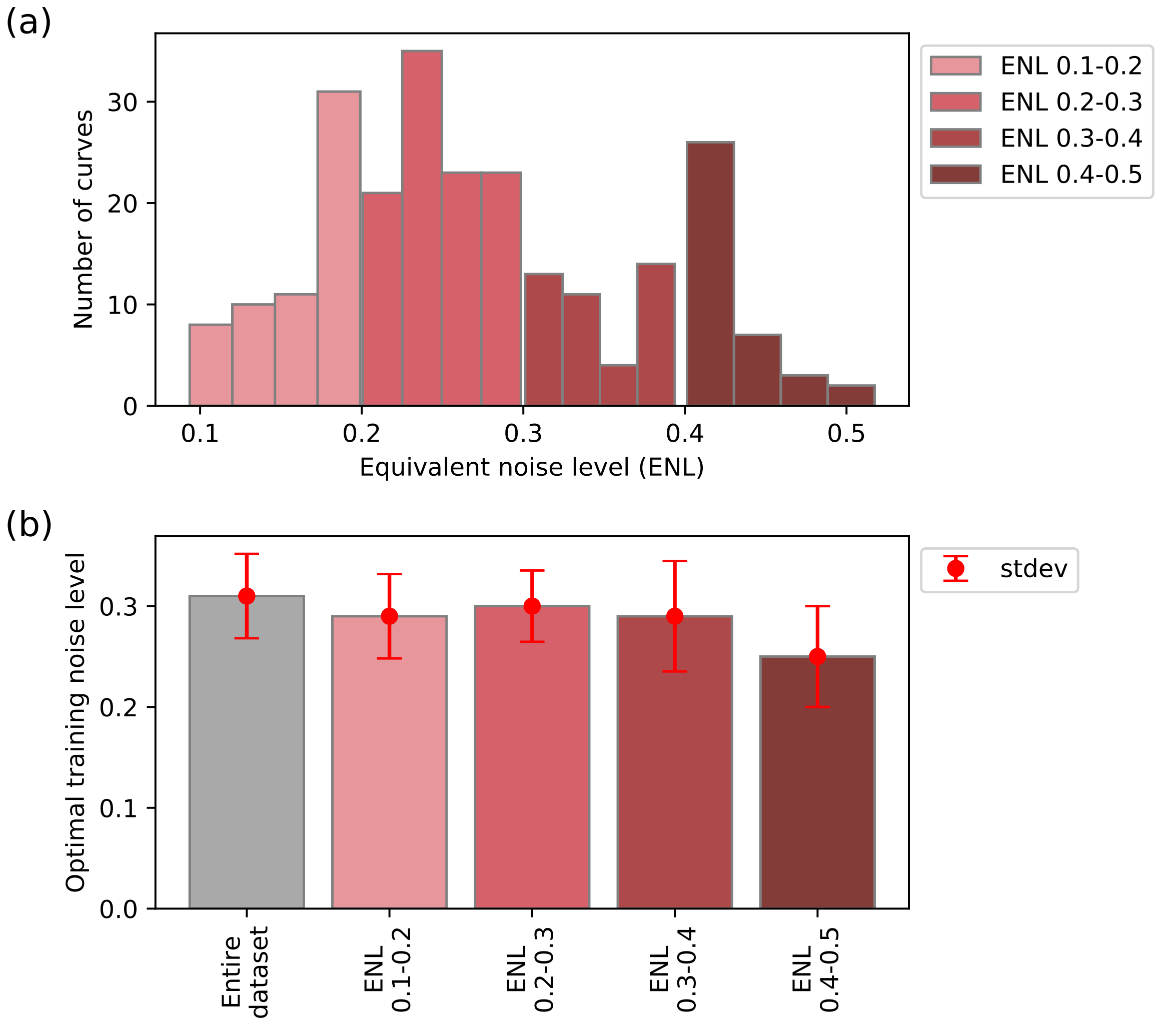

FIG. S1. (a) Distribution of the equivalent noise level (ENL) in the experimental testing dataset of 242 XRR curves. The dataset was split into four categories with varying ENLs to test each separately. (b) Optimal training noise for different ENLs in the training data. The optimal level for the entire dataset corresponds to the minimum shown in Figure 4 of the main manuscript. The error bars represent the standard deviation of five independent training repetitions.

The majority of outliers are due to ambiguous fits (e.g. featureless curves) where multiple parameter combinations lead to a good fit. A common case are very thin films where there are no oscillations visible in the chosen q range.

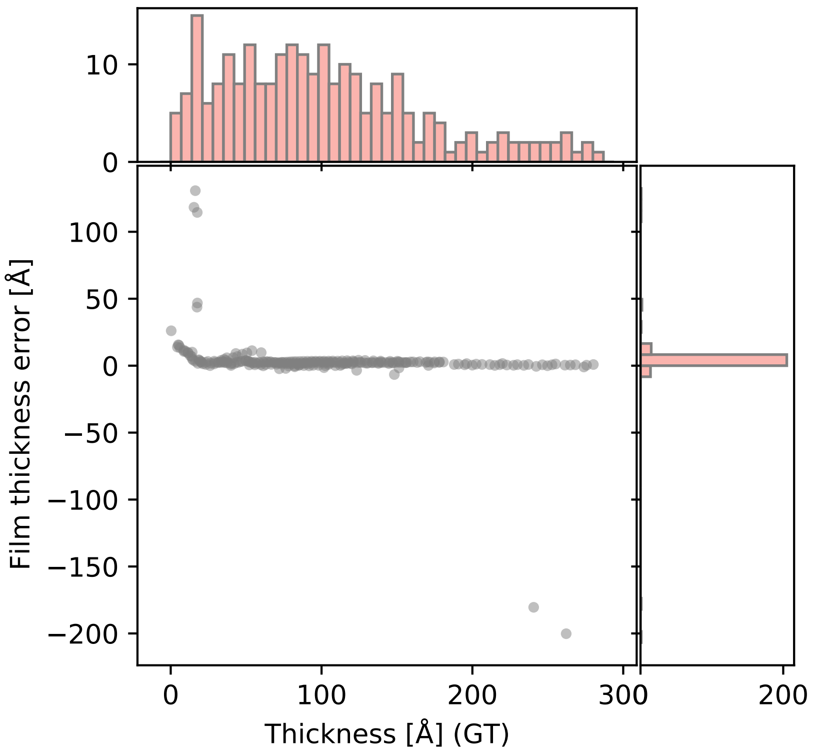

FIG. S2. Distribution of the absolute thickness error from the full pipeline fit with respect to the ground truth (GT) thickness. Each dot represents a single curve in the testing dataset.

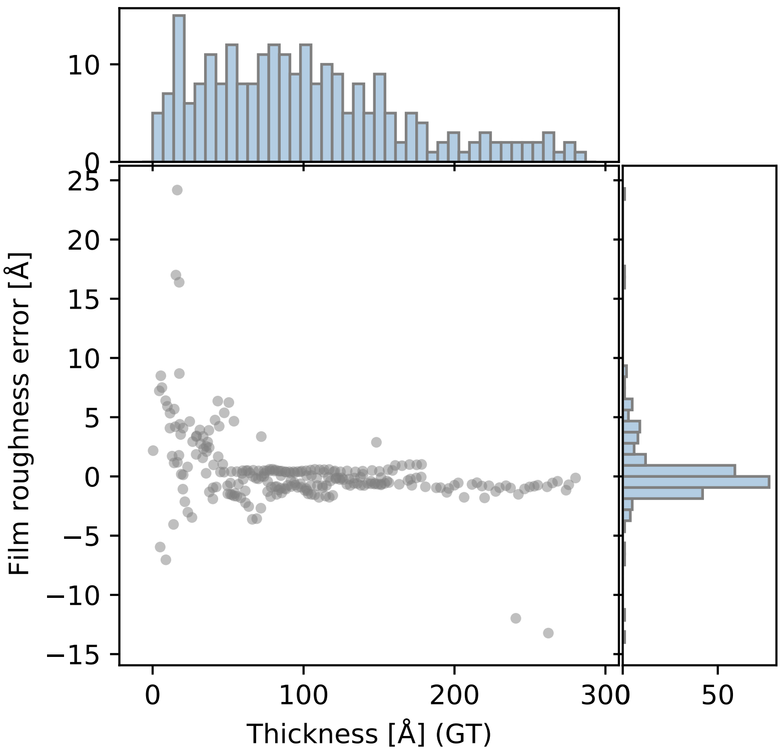

FIG. S3. Distribution of the absolute roughness error from the full pipeline fit with respect to the ground truth (GT) thickness. Each dot represents a single curve in the testing dataset.

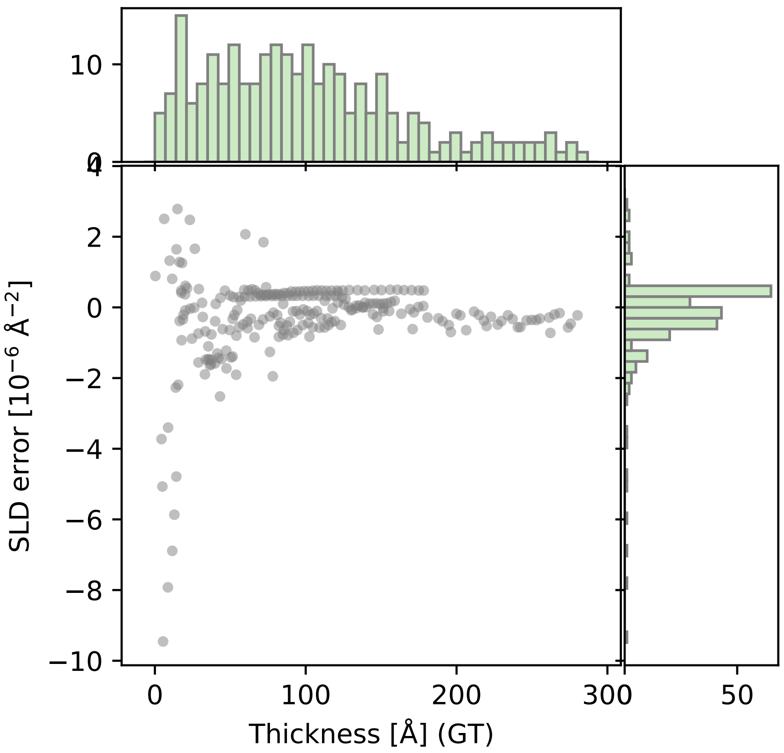

FIG. S4. Distribution of the absolute SLD error from the full pipeline fit with respect to the ground truth (GT) thickness. Each dot represents a single curve in the testing dataset.

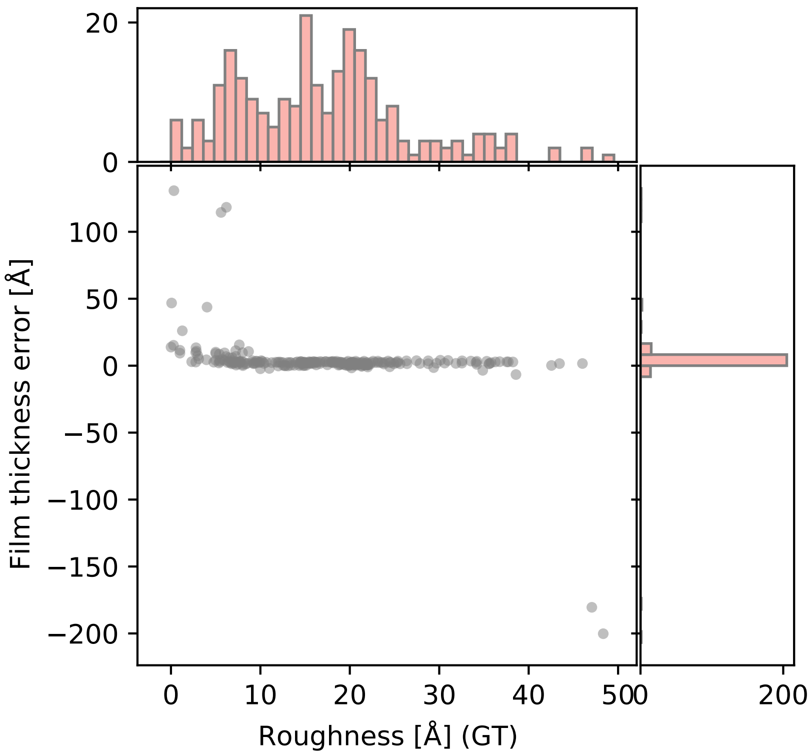

FIG. S5. Distribution of the absolute thickness error from the full pipeline fit with respect to the ground truth (GT) roughness. Each dot represents a single curve in the testing dataset.

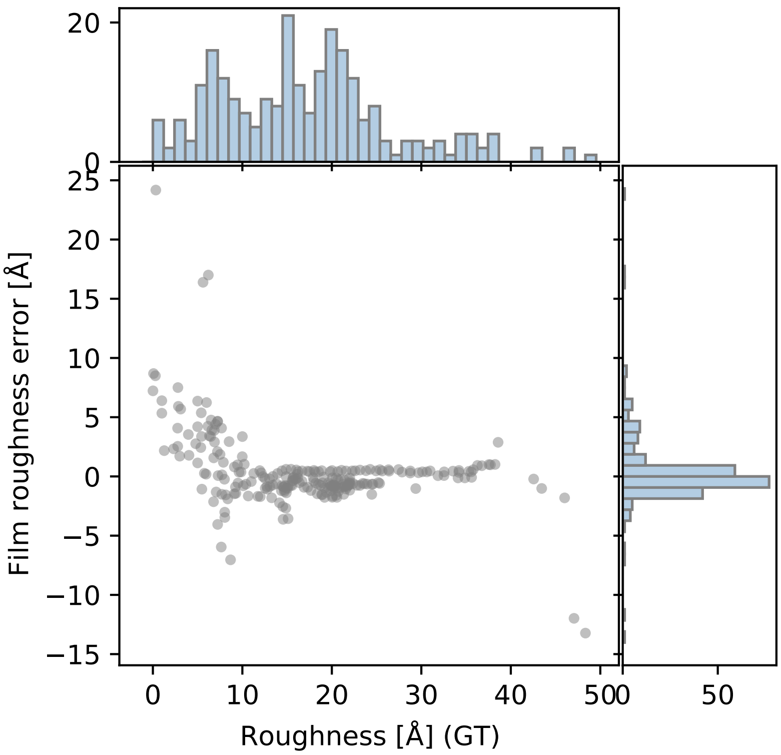

FIG. S6. Distribution of the absolute roughness error from the full pipeline fit with respect to the ground truth (GT) roughness. Each dot represents a single curve in the testing dataset.

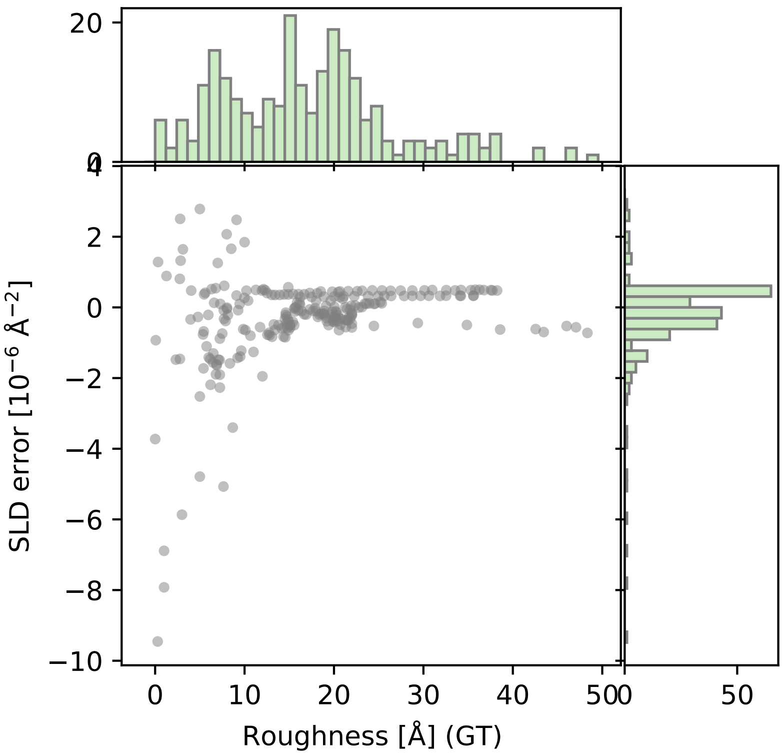

FIG. S7. Distribution of the absolute SLD error from the full pipeline fit with respect to the ground truth (GT) roughness. Each dot represents a single curve in the testing dataset.

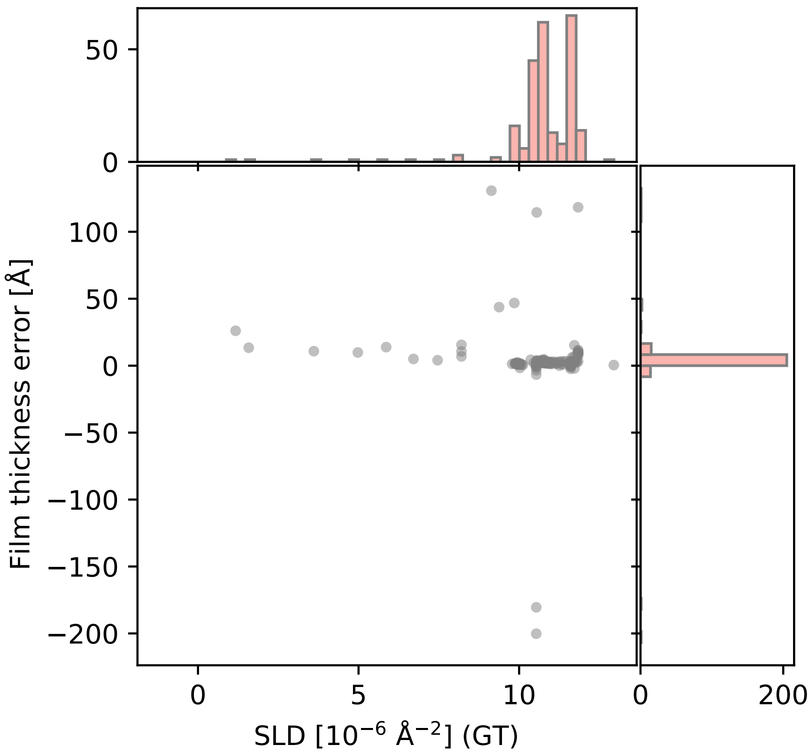

FIG. S8. Distribution of the absolute thickness error from the full pipeline fit with respect to the ground truth (GT) SLD. Each dot represents a single curve in the testing dataset.

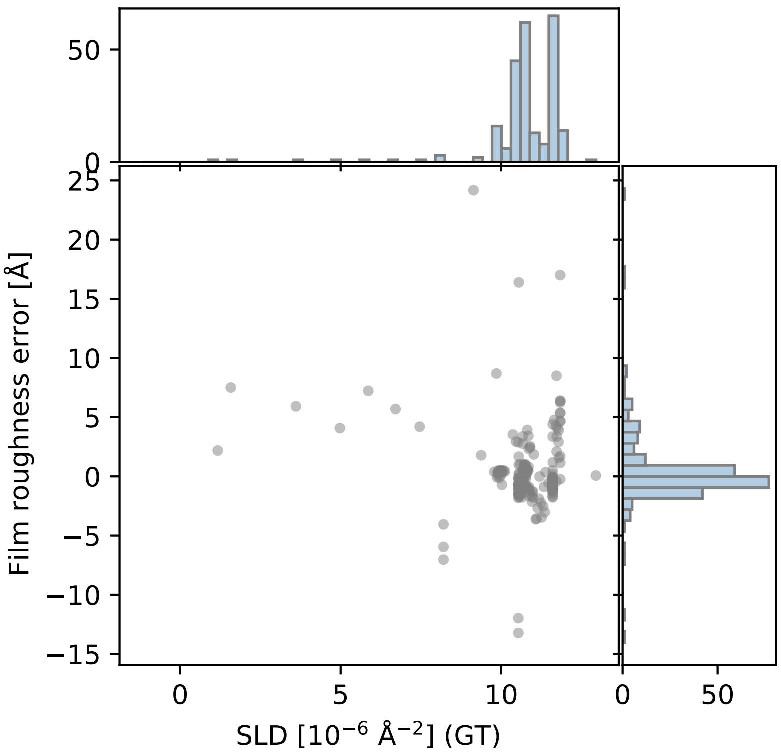

FIG. S9. Distribution of the absolute roughness error from the full pipeline fit with respect to the ground truth (GT) SLD. Each dot represents a single curve in the testing dataset.

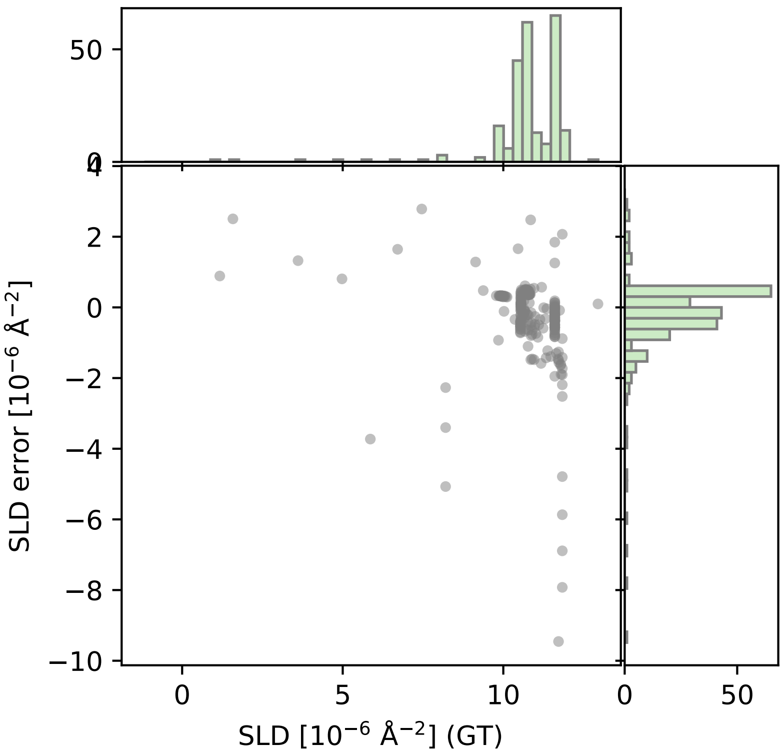

FIG. S10. Distribution of the absolute SLD error from the full pipeline fit with respect to the ground truth (GT) SLD. Each dot represents a single curve in the testing dataset.
