# Supplementary material for: Neural network analysis of neutron and X-ray reflectivity data: automated analysis using mlreflect, experimental errors and feature engineering
Source: J Appl Crystallogr. 2022 Apr 2;55(Pt 2):362–9. doi: 10.1107/S1600576722002230 (PMC8985606; doi:10.1107/S1600576722002230)
Supplement: Supplementary file 2 [file j-55-00362-sup2.zip › examples/pdf/spec_fitter_example.pdf]

spec_fitter_example

January 19, 2022

1 SpecFitter example

This example shows how you can use the pre-trained neural network model to fit a single layer of and organic thin film on a Si/SiOx substrate by directly reading the data from a SPEC file.

[1]:
```
import mlreflect
print('This example was tested with mlreflect version ' + mlreflect.__version__)
```

```
This example was tested with mlreflect version 0.19.0
```

1.1 The SpecFitter class

First we have to import the `SpecFitter` class as well as the path to the example SPEC file. The `DefaultSpecFitter` class is a conventient way to import a `SpecFitter` that already initializes with the aforementioned pre-trained model.

[2]:
```
from mlreflect.curve_fitter import DefaultSpecFitter, example_spec_file_path
```

[3]:
```
spec_fitter = DefaultSpecFitter()
```

We can inspect the sample structure that the model was trained on via the following property:

[4]:
```
print(spec_fitter.trained_model.sample)
```

```
Air (ambient):
        sld: 0 [1e-6 1/Å^2]
[1] Film:
        thickness: (20, 1000) [Å]
        roughness: (0, 100) [Å]
        sld: (1, 14) [1e-6 1/Å^2]
[0] SiOx:
        thickness: 10.0 [Å]
        roughness: 2.5 [Å]
        sld: (17.7735+0.4048j) [1e-6 1/Å^2]
Si (substrate):
        roughness: 1 [Å]
        sld: (20.0701+0.4571j) [1e-6 1/Å^2]
```

It is important to note that the q range that is used for the prediction is fixed after training. The pre-trained model used here was trained for a q range from 0.02 to 0.15 1/Å due to a Bragg peak around 0.3 1/Å.

Next, we must define two dictionaries with important information about the data and the experiment and pass that information to the `SpecFitter` object.

The first `dict` contains the column names of all columns in the SPEC file that together form the full scattering angle (2 theta) and the name of the column where the reflected intensity is saved. You can provide the column name of the attenuator counter with the keyword `attenuator_column`.

The second `dict` contains all parameters that are necessary to apply a basic footprint correction. The wavelength is provided in units of Å. The beam width and sample length along the beam direction are provided in units of mm. By default, the footprint correction assumes a Gaussian profile.

[5]:
```
spec_params = {
    'angle_columns': ['tt'],
    'intensity_column': "p100k_roi2",
    'attenuator_column': 'atten_position'
}

spec_fitter.set_import_params(**spec_params)
```

[6]:
```
footprint_params = {
    'wavelength': 0.6889,
    'beam_width': 0.072,
    'sample_length': 10
}

spec_fitter.set_footprint_params(**footprint_params)
```

Lastly, we need to provide the path the SPEC file that we want to extract the data from. The example dataset provided by the package contains a reflectivity data series from a real-time annealing experiment of a DNTT:PDIF thin film on a Si/SiOx substrate.

[7]:
```
spec_fitter.set_file(example_spec_file_path)
```

```
38 scans found in
/home/greco/git_repos/mlreflect/mlreflect/resources/example/example.spec
```

1.2 Fitting a single curve

The fit of a particular scan on the spec file can be executed *via* the `fit()` method by providing the scan number. When this method is executed, several things happen automatically:

- The data is read from the SPEC file
- Footprint correction is applied
- The data is normalized to 1

- The data is interpolated to the q values required by the neural network input
- The neural network predicts the three thin film parameters (thickness, roughness, SLD)
- A "fit" curve is simulated and plotted with the original data

The fit method takes several optional keywords, some important examples are:

- `trim_front`: This can be important in cases where the direct beam is part of the dataset. In such a case it is recommended to use a value for`trim_front` so that the normalization of the curve is not affected by the direct beam.
- `polish`: If `polish=True`, the result obtained from the neural network is refined by a simple LMS minimization using `scipy.optimize.minimize`. This can sometimes yield a nicer fit, but there is a risk of running into unphysical minima for the roughness or SLD.
- `plot`: If `plot=True` a plot of the curve fit and the SLD profile are shown.

The `fit()` method yields a `FitResult` object that contains all the important results.

[8]:
```
fit_result = spec_fitter.fit(1046, trim_front=3, plot=True, reload=False)
```

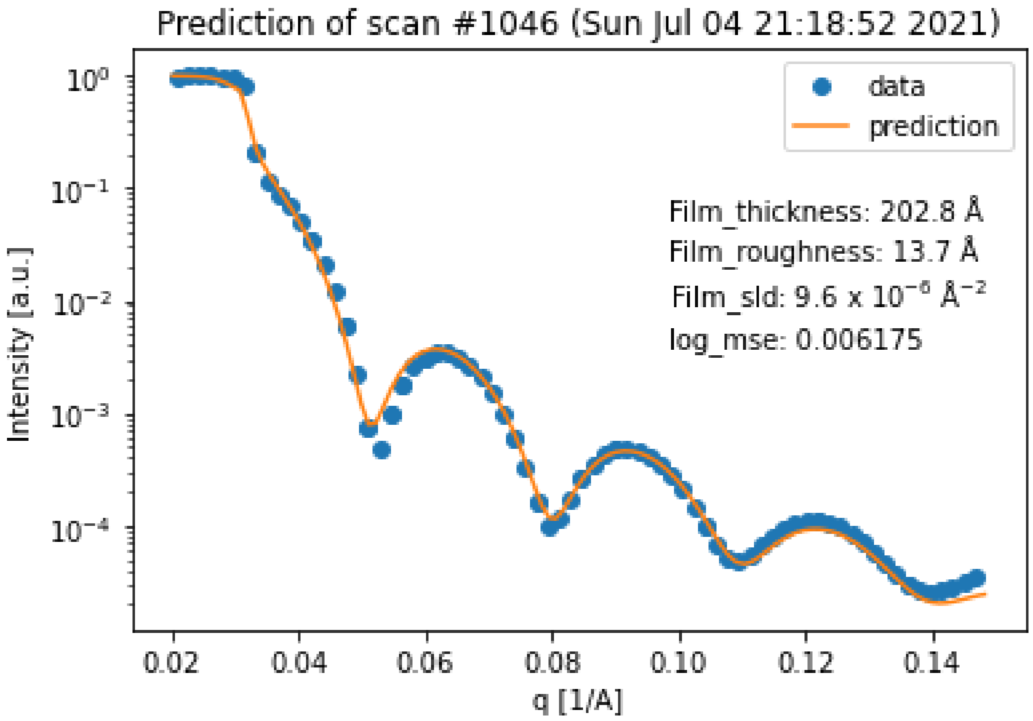

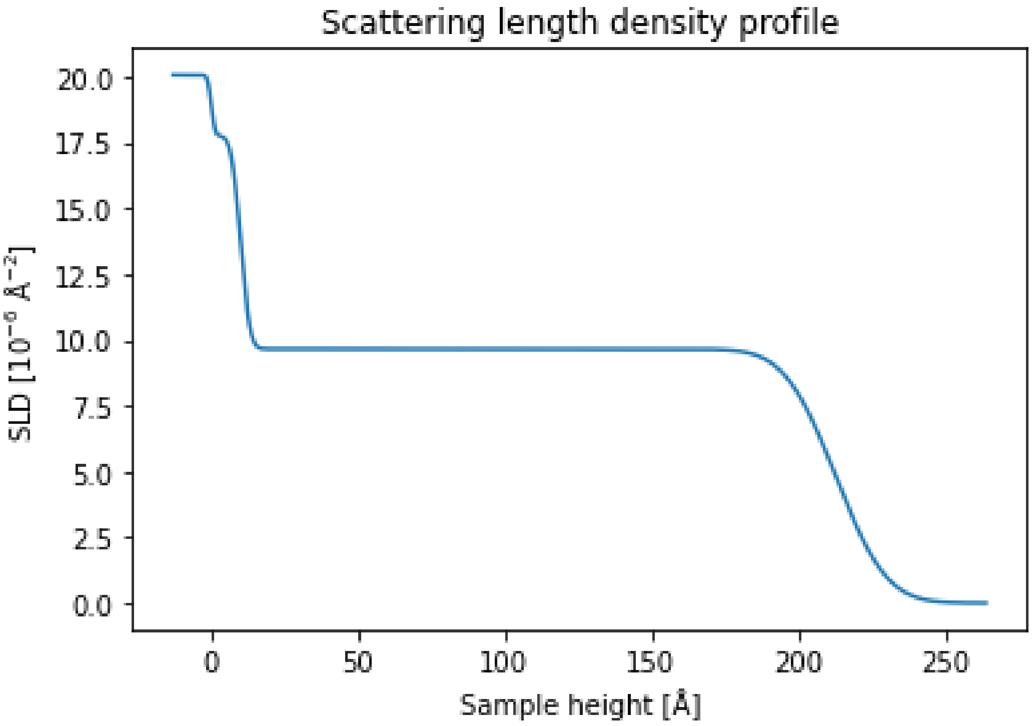

The results are obtained within less than a second and we can see that the guess of the neural network shows a reasonable fit of the data within the given q range.

1.3 Accessing and saving the results

The fit results can also be accessed via the `predicted_parameters` property. This shows the parameter values of the whole box model (including the ones that weren't predicted).

[9]:
```
fit_result.predicted_parameters
```

[9]:
```
      SiOx_thickness  Film_thickness  Si_roughness  SiOx_roughness  \
scan
1046            10.0      202.783216             1             2.5

      Film_roughness             Si_sld            SiOx_sld  Film_sld  \
scan
1046       13.718066  20.070100+0.457100j  17.773500+0.404800j   9.64782

      Air_sld
scan
1046        0
```

The `FitResult` class has several useful methods to save the results as well as the original curve

(including footprint correction).

[10]:
```
fit_result.save_predicted_parameters('parameters.dat')
fit_result.save_predicted_reflectivity('predicted_reflectivity.dat')
fit_result.save_corrected_reflectivity('corrected_reflectivity.dat')
```

1.4 Identifying reflectivity scans

A quick overview of the available scans in the SPEC file can be achieved with the method `SpecFitter.show_scans()`. This is useful to find the scan numbers of reflectivity scans for the `fit()` method.

[11]:
```
spec_fitter.show_scans(min_scan=1046)
```

```
38 scans found in
/home/greco/git_repos/mlreflect/mlreflect/resources/example/example.spec
scan #1046
        command: a2scan om 0.06 0.5 tt 0.12 1.0 79 1.0
        time: Sun Jul 04 21:18:52 2021
scan #1047
        command: dscan hz -0.2 0.2 20 0.1
        time: Sun Jul 04 21:25:42 2021
scan #1048
        command: dscan hz -0.2 0.2 20 0.1
        time: Sun Jul 04 21:30:19 2021
scan #1049
        command: dscan hry -0.05 0.05 20 1.0
        time: Sun Jul 04 21:31:24 2021
scan #1050
        command: dscan hz -0.2 0.2 20 0.1
        time: Sun Jul 04 21:32:52 2021
scan #1051
        command: a2scan om 0.06 0.5 tt 0.12 1.0 79 1.0
        time: Sun Jul 04 21:33:58 2021
scan #1052
        command: dscan hz -0.2 0.2 20 0.1
        time: Sun Jul 04 21:40:59 2021
scan #1053
        command: dscan hry -0.05 0.05 20 1.0
        time: Sun Jul 04 21:42:29 2021
scan #1054
        command: a2scan om 0.06 0.5 tt 0.12 1.0 79 1.0
        time: Sun Jul 04 21:44:38 2021
scan #1055
        command: a2scan om 0.06 0.5 tt 0.12 1.0 79 1.0
        time: Sun Jul 04 21:50:18 2021
scan #1056
```

```
        command: a2scan om 0.06 0.5 tt 0.12 1.0 79 1.0
        time: Sun Jul 04 21:55:52 2021
scan #1057
        command: a2scan om 0.06 0.5 tt 0.12 1.0 79 1.0
        time: Sun Jul 04 22:01:18 2021
scan #1058
        command: a2scan om 0.06 0.5 tt 0.12 1.0 79 1.0
        time: Sun Jul 04 22:06:46 2021
scan #1059
        command: dscan hz -0.2 0.2 20 0.1
        time: Sun Jul 04 22:15:56 2021
```

1.5 Fitting a range of curves

In order to analyze a whole series of scans at once (which is useful for a temperature series such as this one) we can use the `fit_range()` method. It works almost the exact same as the `fit()` method except that we have to provide a list of scan numbers and that it yields a `FitResultSeries` object.

[12]:
```
scan_numbers = [1046, 1051] + list(range(1054, 1059))
print(scan_numbers)
```

```
[1046, 1051, 1054, 1055, 1056, 1057, 1058]
```

[13]:
```
fit_result_series = spec_fitter.fit_range(scan_numbers, trim_front=3,␣
 ↪plot=True, reload=False)
```

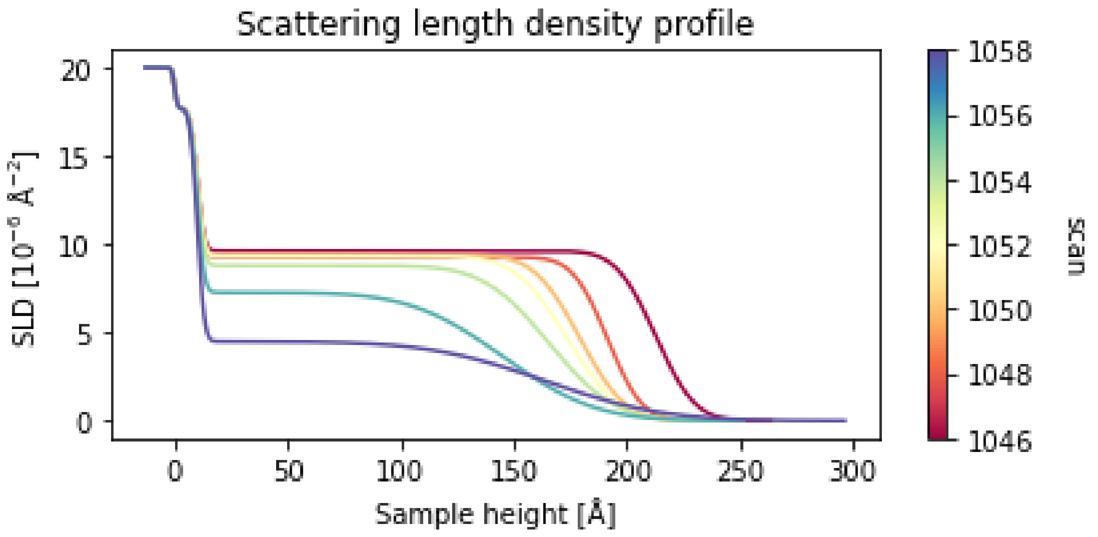

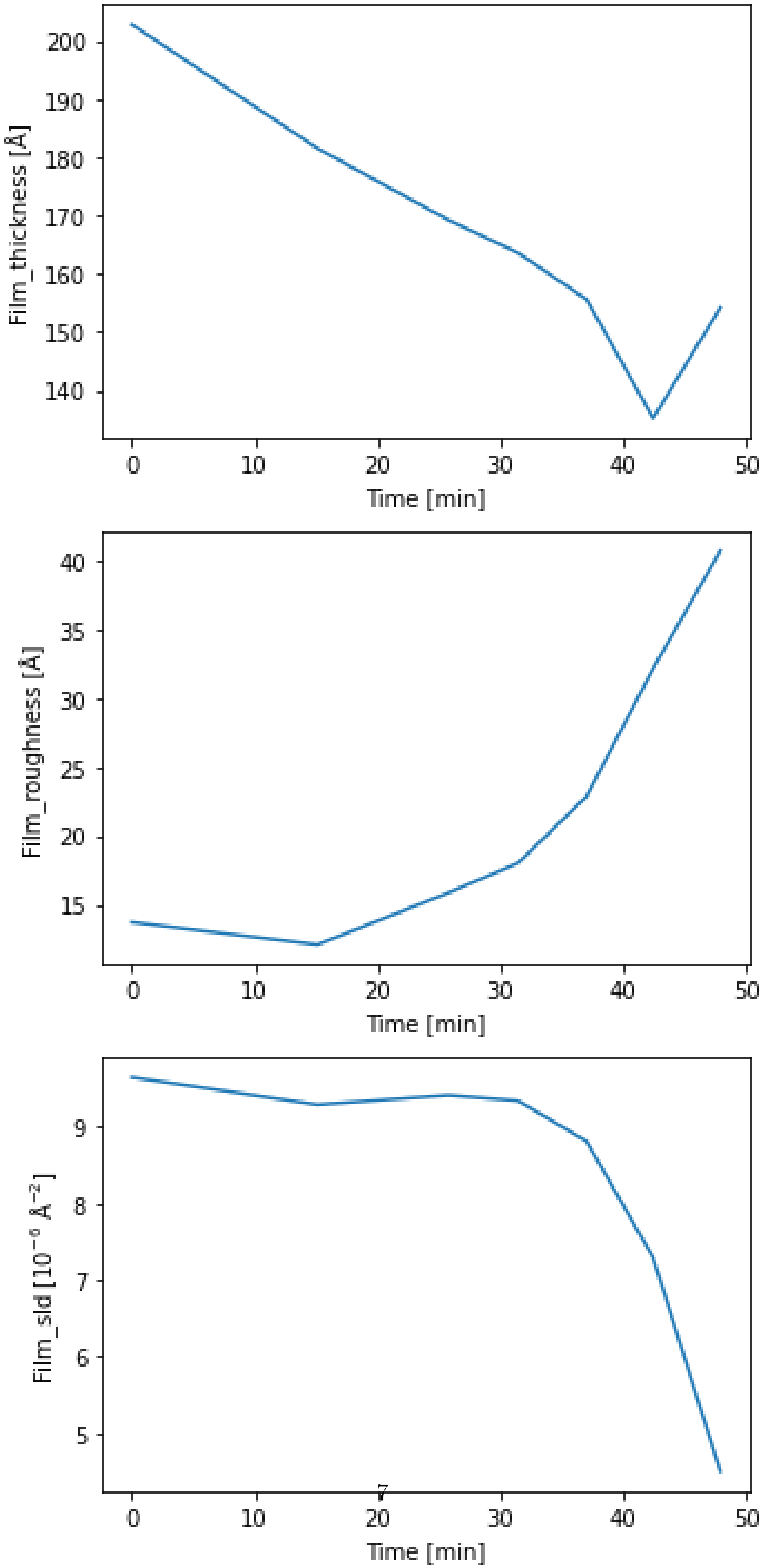
Film_thickness [Å]
200
190
180
170
160
150
140
0
10
20
30
40
50
Time [min]
Film_roughness [Å]
40
35
30
25
20
15
0
10
20
30
40
50
Time [min]
Film_sld [10^{-6} Å^{-2}]
9
8
7
6
5
0
10
20
30
40
50
Time [min]

From the plot output we can a nice trend in the data which shows that the film becomes thinner and a lot rougher during the temperature ramp, indicating the expected desorption of the film.

If we want to inspect every single fit of the neural network, we can use the `fit_results_list` property to access every individual `FitResult` object.

[14]:
```
for result in fit_result_series.fit_results_list:
    result.plot_prediction(['Film_thickness', 'Film_roughness', 'Film_sld'])
```

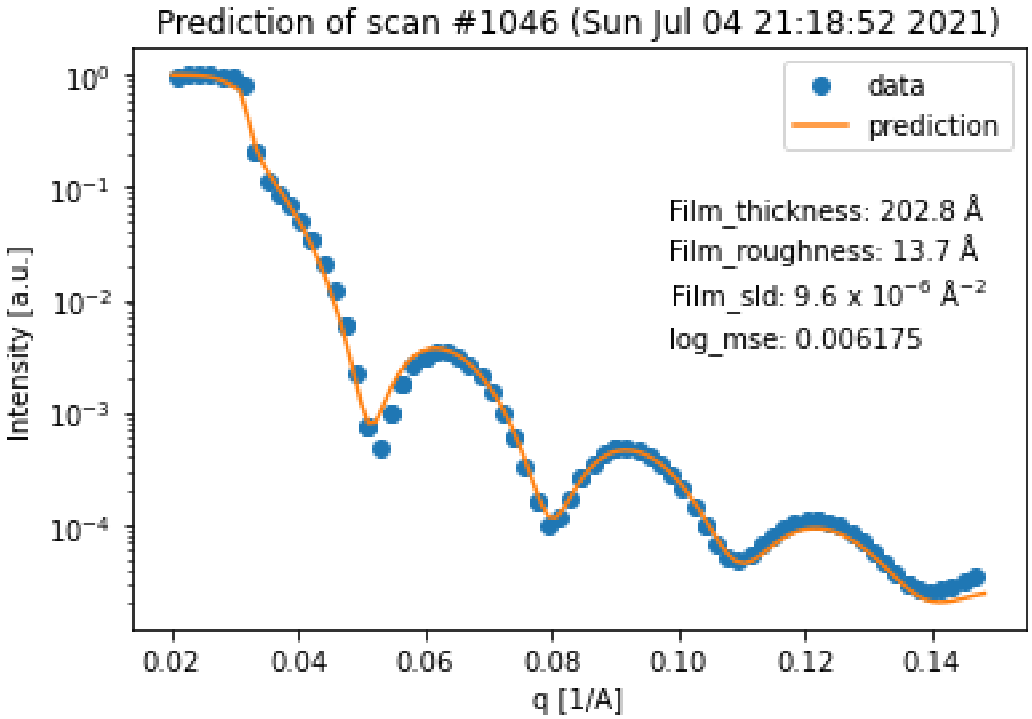

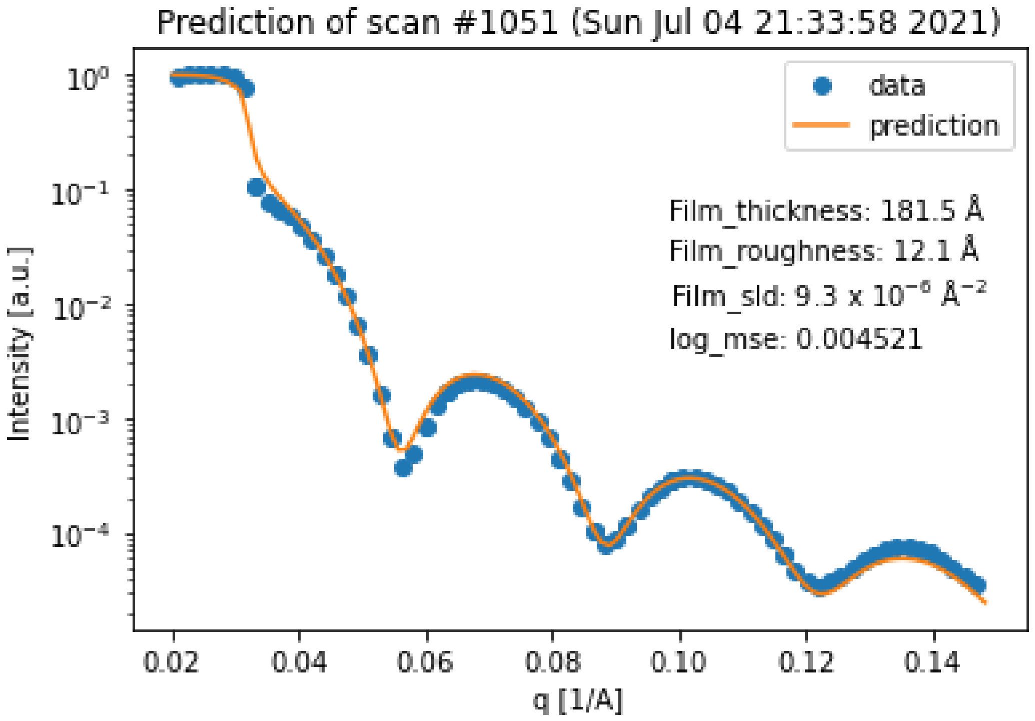
Prediction of scan #1051 (Sun Jul 04 21:33:58 2021)
data
prediction
Film_thickness: 181.5 Å
Film_roughness: 12.1 Å
Film_sld: 9.3 x 10⁻⁶ Å⁻²
log_mse: 0.004521
Intensity [a.u.]
q [1/A]

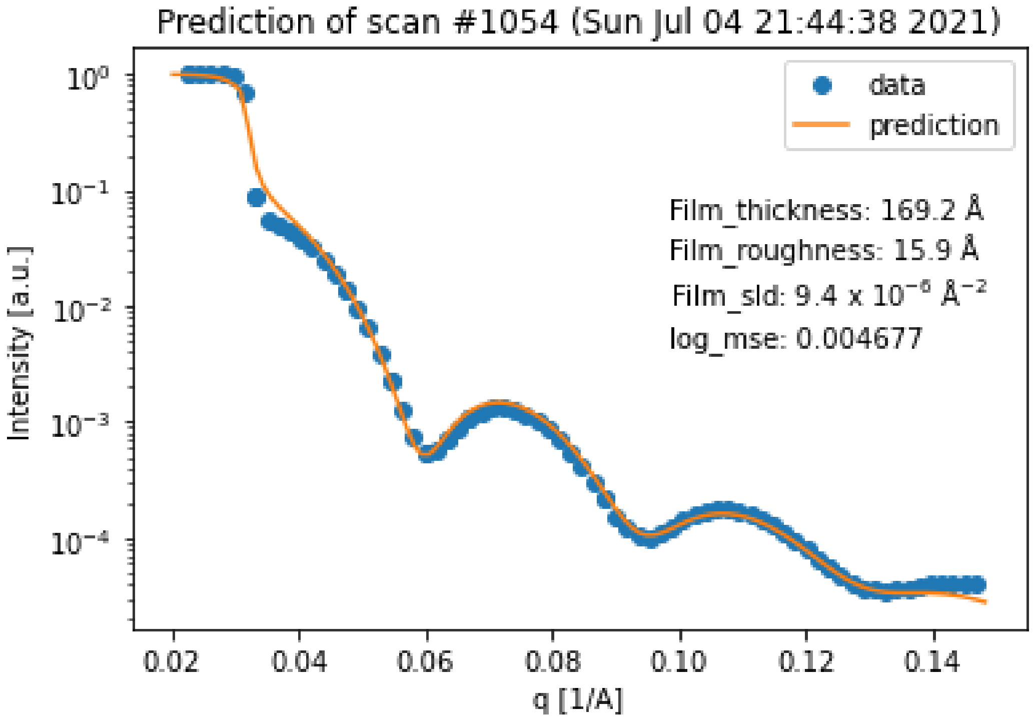
Prediction of scan #1054 (Sun Jul 04 21:44:38 2021)
data
prediction
Film_thickness: 169.2 Å
Film_roughness: 15.9 Å
Film_sld: 9.4 x 10⁻⁶ Å⁻²
log_mse: 0.004677
Intensity [a.u.]
q [1/A]

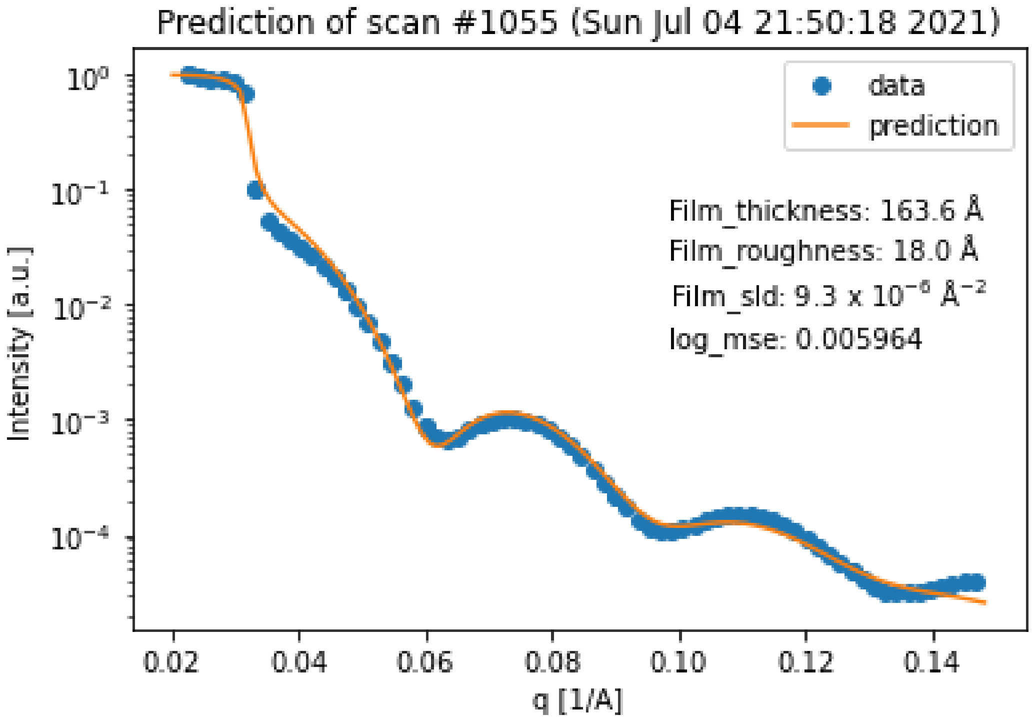
Prediction of scan #1055 (Sun Jul 04 21:50:18 2021)
data
prediction
Film_thickness: 163.6 Å
Film_roughness: 18.0 Å
Film_sld: 9.3 x 10⁻⁶ Å⁻²
log_mse: 0.005964
Intensity [a.u.]
q [1/A]

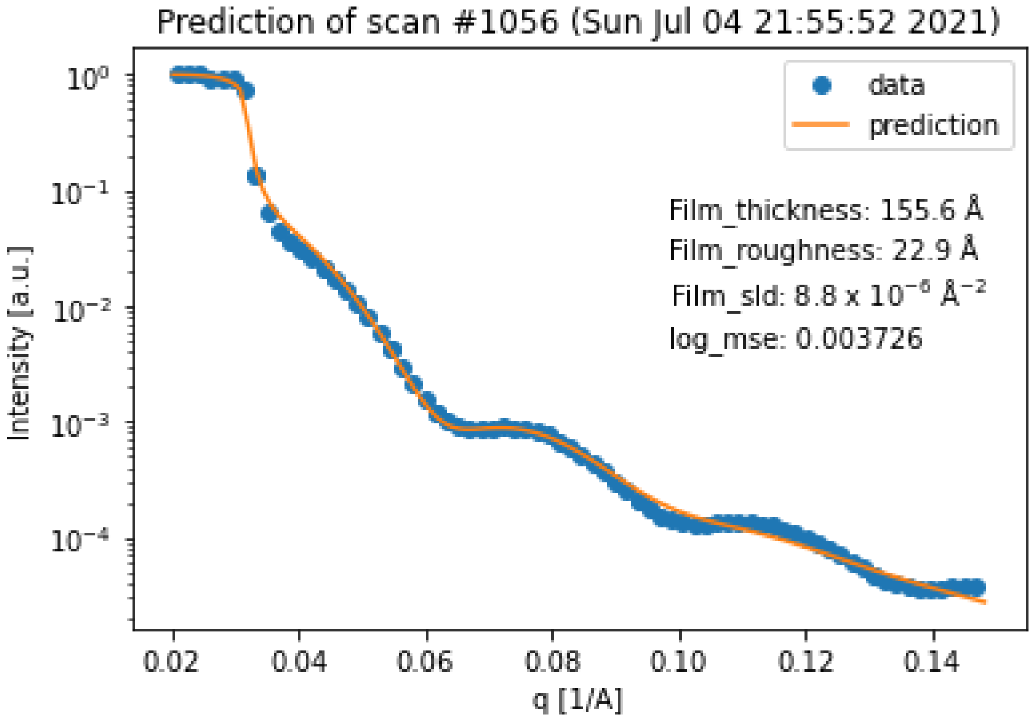
Prediction of scan #1056 (Sun Jul 04 21:55:52 2021)
data
prediction
Film_thickness: 155.6 Å
Film_roughness: 22.9 Å
Film_sld: 8.8 x 10⁻⁶ Å⁻²
log_mse: 0.003726
Intensity [a.u.]
q [1/A]

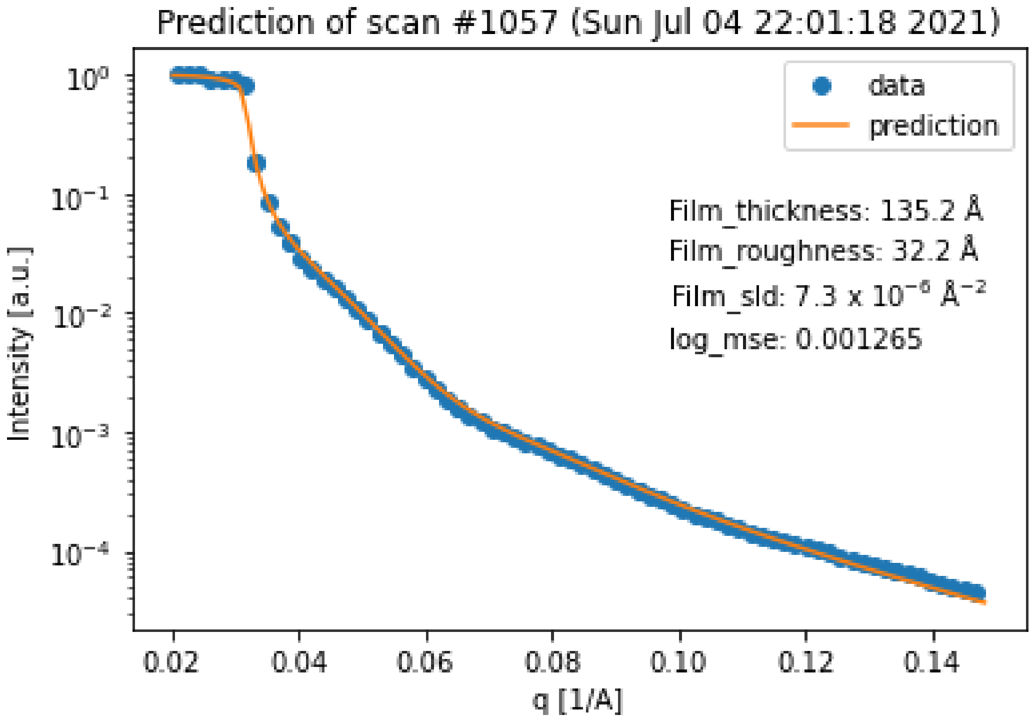
Prediction of scan #1057 (Sun Jul 04 22:01:18 2021)
data
prediction
Film_thickness: 135.2 Å
Film_roughness: 32.2 Å
Film_sld: 7.3 x 10⁻⁶ Å⁻²
log_mse: 0.001265
Intensity [a.u.]
q [1/A]

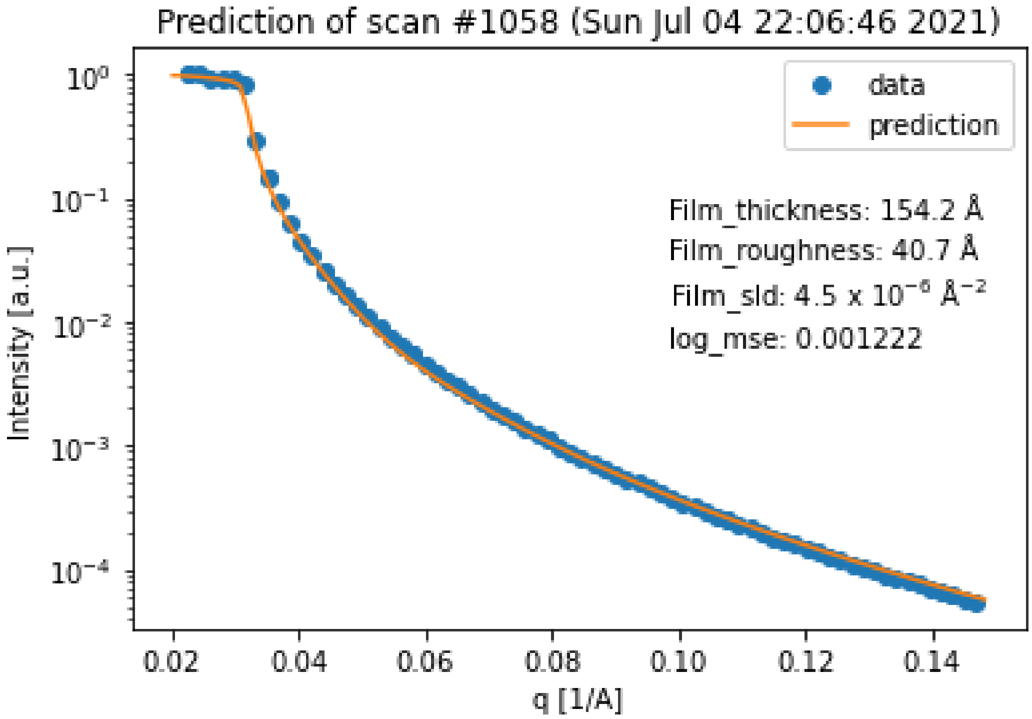
Prediction of scan #1058 (Sun Jul 04 22:06:46 2021)
data
prediction
Film_thickness: 154.2 Å
Film_roughness: 40.7 Å
Film_sld: 4.5 x 10⁻⁶ Å⁻²
log_mse: 0.001222
Intensity [a.u.]
q [1/A]

Again, we can see that all of the fits are quite good. However, in terms of the thickness trend, the last curve seems to be an outlier. This can be explained by the fact that the last curve has almost no features anymore, which makes it very difficult to extract any information (especially about the thickness) from it.
