# Supplementary material for: Neural network analysis of neutron and X-ray reflectivity data: automated analysis using mlreflect, experimental errors and feature engineering
Source: J Appl Crystallogr. 2022 Apr 2;55(Pt 2):362–9. doi: 10.1107/S1600576722002230 (PMC8985606; doi:10.1107/S1600576722002230)
Supplement: Supplementary file 2 [file j-55-00362-sup2.zip › examples/pdf/training_example.pdf]

training_example

January 19, 2022

1 Neural network training example

This examples shows how to train a neural network model for a given sample structure and q range.

[1]:
```
import mlreflect
print('Tested with mlreflect version: ' + mlreflect.__version__)
```

```
Tested with mlreflect version: 0.19.0
```

Whether or not a GPU is available for training can be checked with this function:

[2]:
```
from mlreflect.utils import check_gpu
check_gpu() # Checks if a GPU on the current machine is available
```

```
[PhysicalDevice(name='/physical_device:GPU:0', device_type='GPU')]
```

If the list is empty, either there is no valid GPU or CUDA has not been set up properly. However, training is still possible on CPUs, albeit a bit slower.

1.1 Loading experimental data

Here we first load some template dataset which contains the q values for which we want to train our model as well as experimental data which we can use for testing later.

The example data contains columns of reflectivity data of a time series of a growing DIP thin film on a Si/SiOx substrate with the first column containing the q values in units 1/Å.

[3]:
```
import matplotlib.pyplot as plt
import numpy as np
from mlreflect.curve_fitter import example_ascii_file_path
```

[4]:
```
experiment = np.loadtxt(example_ascii_file_path)
```

[5]:
```
q = experiment[:, 0] # Extract q values
experimental_reflectivity = experiment[:, 1:].T # Extract intensity values
```

[6]:
```
plt.semilogy(q, experimental_reflectivity[30, :], '.')
plt.xlabel('q [1/A]')
plt.ylabel('Reflectivity [norm.]')
```

```
plt.title('Example reflectivity curve')
plt.show()
```

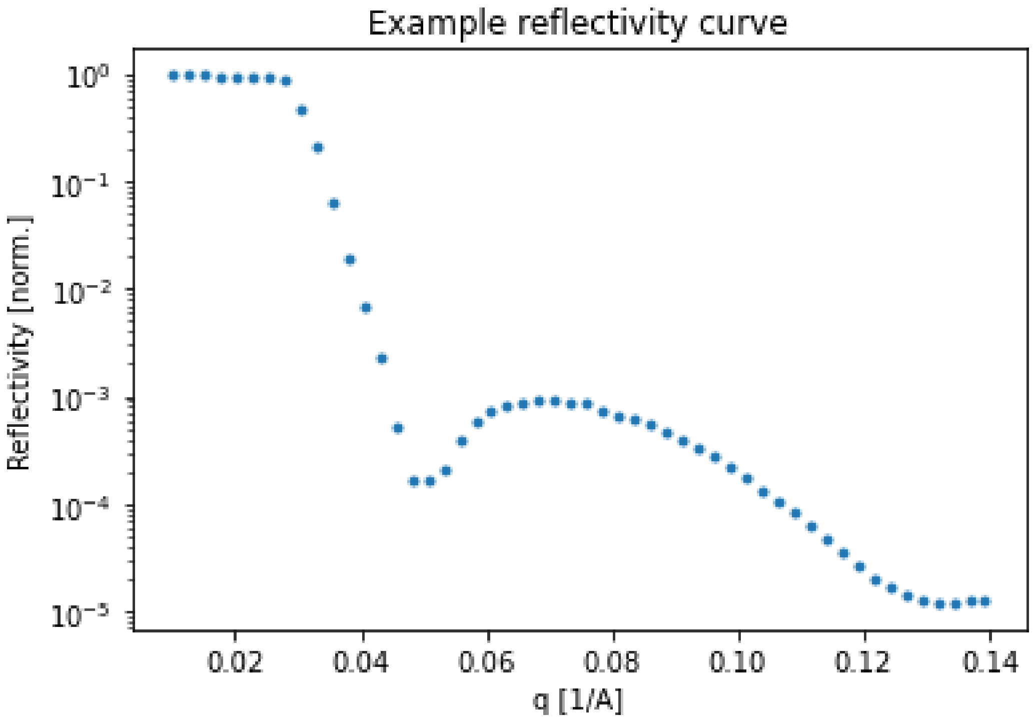

1.2 Generating training data training data

Before generating the training data, a appropriate box model for the sample has to be chosen. The subpackage `mlreflect.data_generation` contains several classes for that.

[7]:
```
from mlreflect.data_generation import Layer, Substrate, AmbientLayer,␣
↪MultilayerStructure
```

[8]:
```
substrate = Substrate('Si', 1, 20.0701+0.4571j)
layer1 = Layer('SiOx', 10, 2.5, 17.7735+0.4048j)
layer2 = Layer('Film', (20, 300), (0, 60), (1, 14))
ambient = AmbientLayer('ambient', 0)
```

The layers are then added to a `MultilayerStructure` object. A `MultilayerStructure` object can have any number of layers, but must always have a substrate and an ambient layer.

[9]:
```
sample = MultilayerStructure()
sample.set_substrate(substrate)
sample.set_ambient_layer(ambient)
sample.add_layer(layer1)
```

```
sample.add_layer(layer2)
```

[10]:
```
print(sample)
```

```
ambient (ambient):
        sld: 0 [1e-6 1/Å^2]
[1] Film:
        thickness: (20, 300) [Å]
        roughness: (0, 60) [Å]
        sld: (1, 14) [1e-6 1/Å^2]
[0] SiOx:
        thickness: 10 [Å]
        roughness: 2.5 [Å]
        sld: (17.7735+0.4048j) [1e-6 1/Å^2]
Si (substrate):
        roughness: 1 [Å]
        sld: (20.0701+0.4571j) [1e-6 1/Å^2]
```

1.3 Training the neural network

The `Trainer` object is used for generating training data and training a neural network of a pre-defined architecture. The `Trainer` object requires the sample structure we just defined as well as the q values.

The training data conists of random parameter sets (called labels) as well as their corresponding reflectivity simulations.

[11]:
```
from mlreflect.training import Trainer
```

[12]:
```
trainer = Trainer(sample, q, random_seed=10)
```

Training data is generated `generate_training_data()` method,

[13]:
```
trainer.generate_training_data(2**14)
```

```
100%|          | 16384/16384 [00:00<00:00, 17958.71it/s]
```

[14]:
```
trainer.training_data['labels'].head(5) # Quick inspection if the generated␣
 ↪parameters are as expected
```

[14]:
```
   SiOx_thickness  Film_thickness  Si_roughness  SiOx_roughness  \
0              10      262.539640             1             2.5
1              10       71.453799             1             2.5
2              10       20.795852             1             2.5
3              10       21.603034             1             2.5
4              10      256.047506             1             2.5

   Film_roughness                Si_sld           SiOx_sld   Film_sld  \
```

```
0       50.998628  20.070100+0.457100j  17.773500+0.404800j  13.619054
1        4.411697  20.070100+0.457100j  17.773500+0.404800j   1.554107
2        2.066177  20.070100+0.457100j  17.773500+0.404800j  10.526338
3        8.667772  20.070100+0.457100j  17.773500+0.404800j   8.482257
4        1.420925  20.070100+0.457100j  17.773500+0.404800j  12.064421

   ambient_sld
0            0
1            0
2            0
3            0
4            0
```

Optionally, you can use the `ReflectivityGenerator` class to generate reflectivity curves and SLD profiles from the parameter sets in the training data.

[15]:
```
from mlreflect.data_generation import ReflectivityGenerator
```

[16]:
```
generator = ReflectivityGenerator(q, sample)
```

[17]:
```
sld_profiles = generator.simulate_sld_profiles(trainer.training_data['labels'].
 ↪head(5))
for i in range(5):
    plt.plot(sld_profiles[i][0], sld_profiles[i][1])

plt.xlabel('sample height [A]')
plt.ylabel('SLD [1e-6 1/A$^2$]')
plt.title('SLD profiles')
plt.show()
```

```
100%|     | 5/5 [00:00<00:00, 2560.00it/s]
```

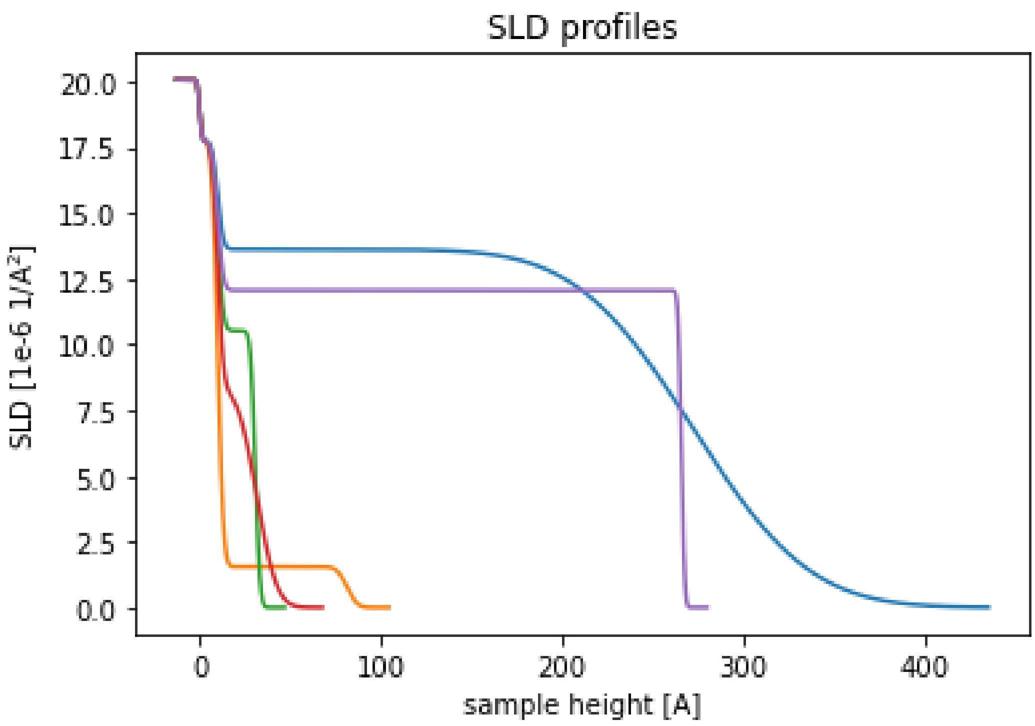

[18]:
```
for i in range(5):
    plt.semilogy(q, trainer.training_data['reflectivity'][i, :], label=str(i))
plt.xlabel('q [1/A]')
plt.ylabel('Reflectivity [norm.]')
plt.legend()
plt.show()
```

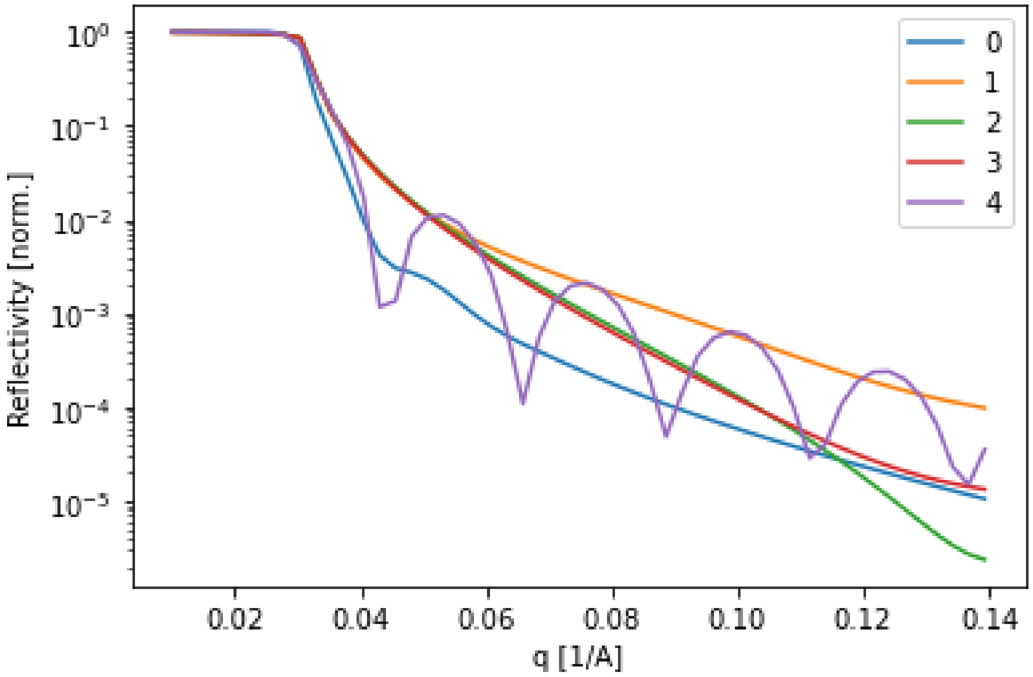

The training is started *via* the `train()` method. It requires a number of epochs for training and a batch size. If unsure what to choose, the default vales will probably work (although it may take longer).

[19]:
```
trained_model, hist = trainer.train(n_epochs=80, batch_size=512, verbose=0)
```

```
Epoch 00045: ReduceLROnPlateau reducing learning rate to 0.0005000000237487257.

Epoch 00065: ReduceLROnPlateau reducing learning rate to 0.0002500000118743628.

Epoch 00078: ReduceLROnPlateau reducing learning rate to 0.0001250000059371814.
Time needed for training: 0:00:06.774026
```

The training and validation loss can be inspected via the `history` dictionary. If the losses have not reached saturation, the training might have been too short. If the validation loss is much higher than the training loss, there might be an overfitting problem.

[20]:
```
plt.plot(hist.history['loss'], label='loss')
plt.plot(hist.history['val_loss'], label='val_loss')
plt.xlabel('Epoch')
plt.ylabel('Loss')
plt.title('Loss (linear)')
plt.legend()
plt.show()
```

```
plt.semilogy(hist.history['loss'], label='loss')
plt.semilogy(hist.history['val_loss'], label='val_loss')
plt.xlabel('Epoch')
plt.ylabel('Loss')
plt.title('Loss (log)')
plt.legend()
plt.show()
```

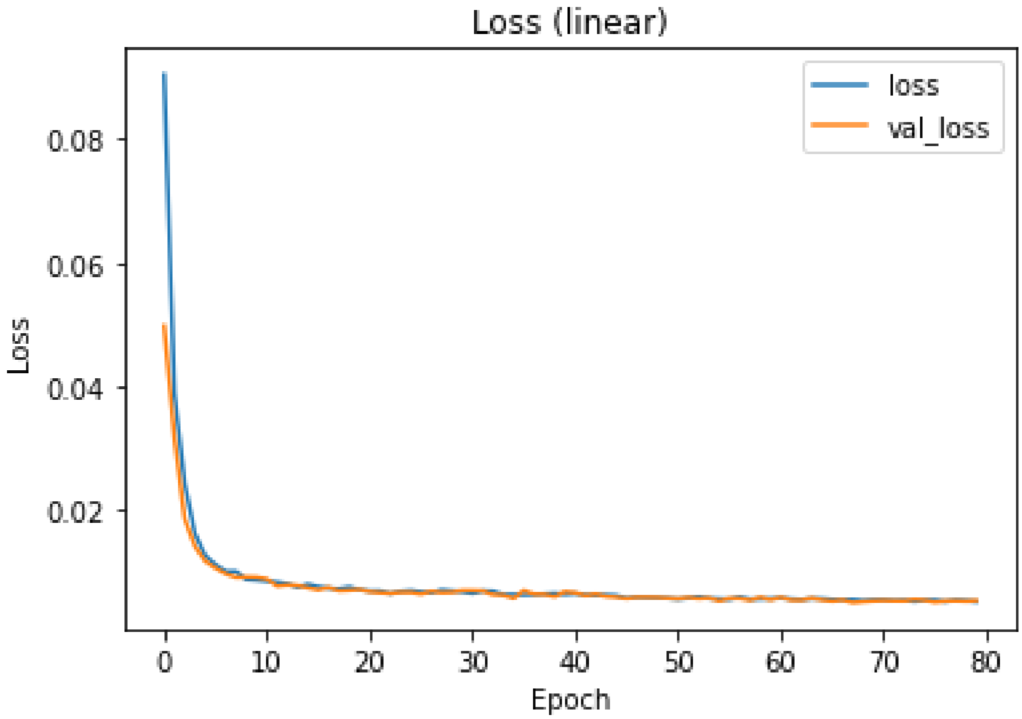

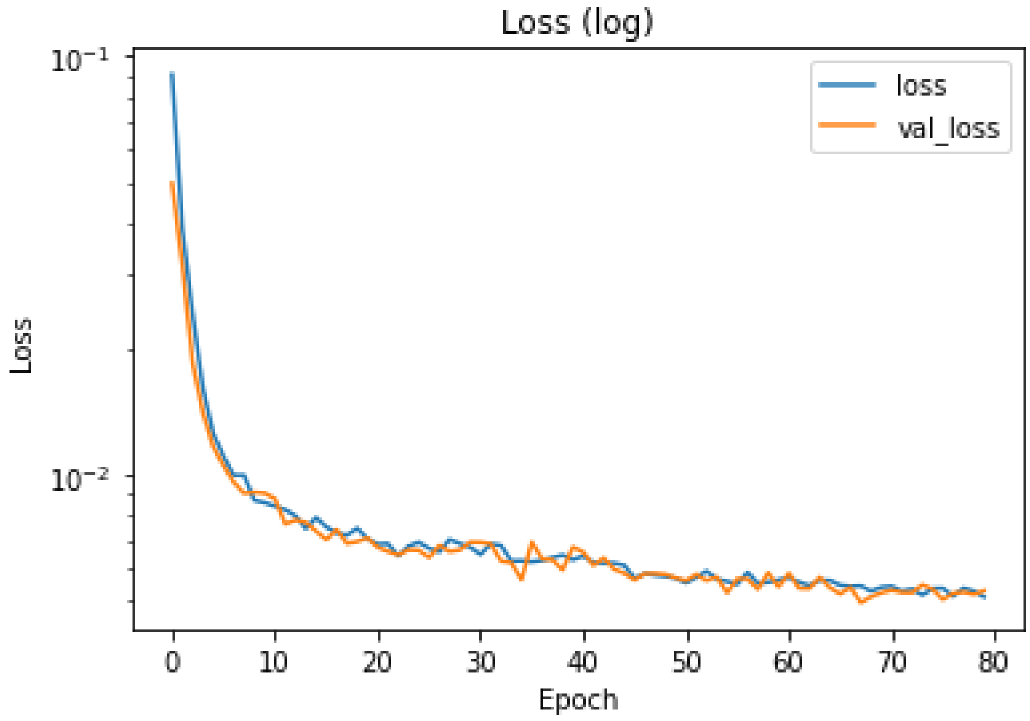

1.4 The CurveFitter class

1.4.1 Testing on simulated data

Using the `TrainedModel` output from the `train()` method, we can initialize a `CurveFitter` to predict thin film parameters on simulated test data.

[21]:
```
from mlreflect.curve_fitter import CurveFitter
```

[22]:
```
curve_fitter = CurveFitter(trained_model)
```

[23]:
```
test_labels = generator.generate_random_labels(2**13)
test_reflectivity = generator.simulate_reflectivity(test_labels)
```

```
100%|     | 8192/8192 [00:00<00:00, 18405.90it/s]
```

We can fit the test data with the `fit_curve()` method, which takes an `ndarray` of reflectivity curves (with each row being one curve) and a list of q values for each data point. We turn the keywowrds `polish` and `optimize_q` off, because they are part of the post-processing step and are not part of the performance evaluation of the neural network model itself.

[24]:
```
fit_output = curve_fitter.fit_curve(test_reflectivity, q, polish=False,␣
↪optimize_q=False)
```

[25]:
```
predicted_test_labels = fit_output['predicted_parameters']
predicted_test_reflectivity = fit_output['predicted_reflectivity']
```

If we plot the predicted parameters against the true parameters, we can see that most of the predictions have a very low error (although there are some outliers).

[34]:
```
plt.plot(predicted_test_labels['Film_thickness'],␣
 ↪test_labels['Film_thickness'], '.', label='predicted', alpha=0.1)
plt.plot(predicted_test_labels['Film_thickness'],␣
 ↪predicted_test_labels['Film_thickness'], label='ground truth')
plt.legend()
plt.xlabel('Ground truth')
plt.ylabel('Thickness')
plt.show()

plt.plot(predicted_test_labels['Film_roughness'],␣
 ↪test_labels['Film_roughness'], '.', label='predicted', alpha=0.1)
plt.plot(predicted_test_labels['Film_roughness'],␣
 ↪predicted_test_labels['Film_roughness'], label='ground truth')
plt.legend()
plt.xlabel('Ground truth')
plt.ylabel('Roughness')
plt.show()

plt.plot(predicted_test_labels['Film_sld'], test_labels['Film_sld'], '.',␣
 ↪label='predicted', alpha=0.1)
plt.plot(predicted_test_labels['Film_sld'], predicted_test_labels['Film_sld'],␣
 ↪label='ground truth')
plt.legend()
plt.xlabel('Ground truth')
plt.ylabel('SLD')
plt.show()
```

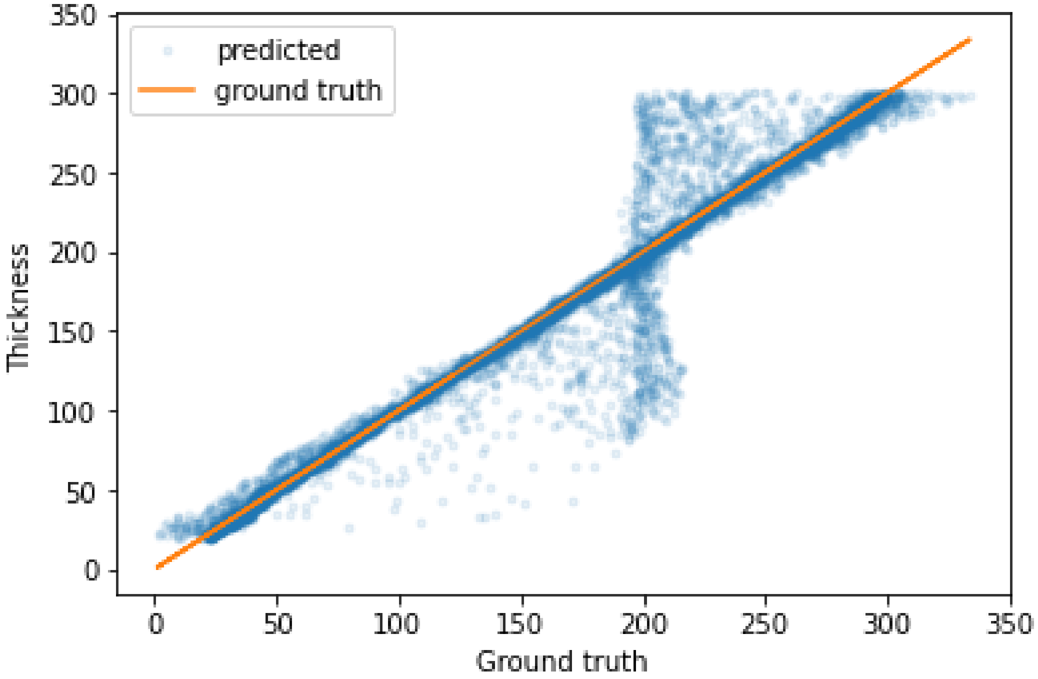
predicted
ground truth
Thickness
Ground truth
0
50
100
150
200
250
300
350

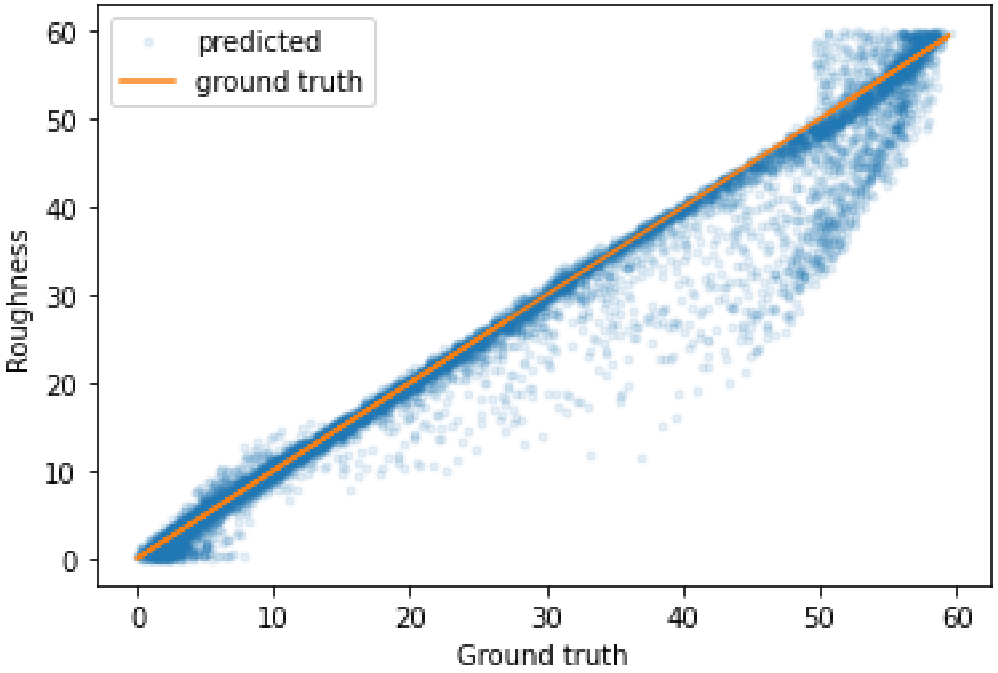
predicted
ground truth
Roughness
Ground truth
0
10
20
30
40
50
60

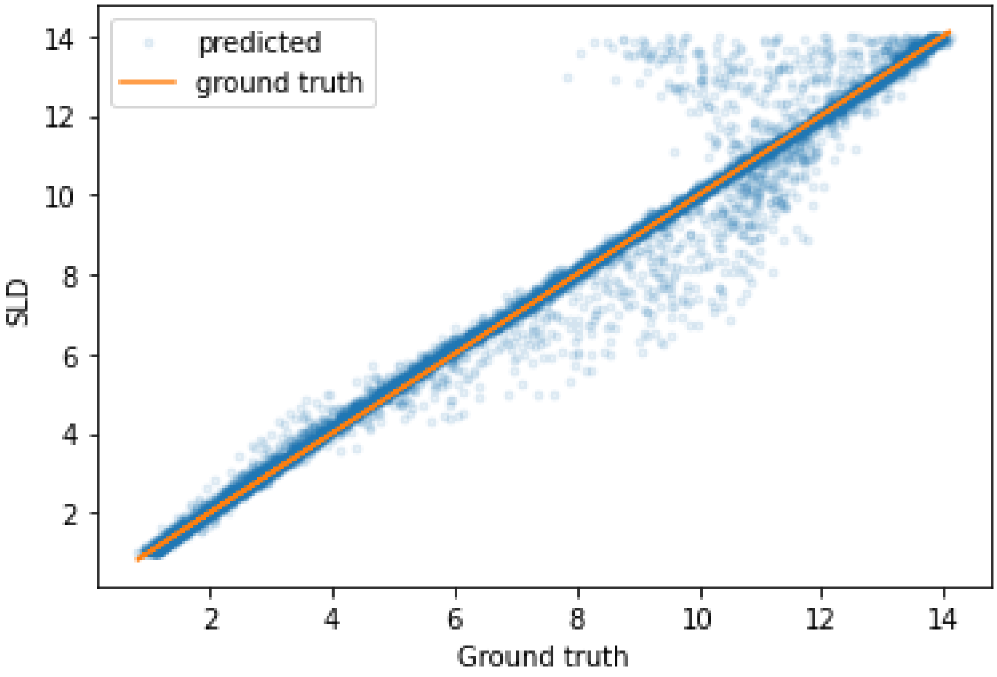

We can also calculate the mean error for each parameter and indeed it is quite low.

[32]:
```
abs(predicted_test_labels - test_labels).mean()
```

[32]:
```
SiOx_thickness     0.000000
Film_thickness     9.078482
Si_roughness       0.000000
SiOx_roughness     0.000000
Film_roughness     1.885550
Si_sld             0.000000
SiOx_sld           0.000000
Film_sld           0.251353
ambient_sld        0.000000
dtype: float64
```

Lastly, we can check some of the curves and compare the fit of the predicted curve. Again, most of the curves seem to be fitted very well.

[47]:
```
for i in np.random.uniform(low=0, high=8475, size=3).astype(int):
    plt.semilogy(q, test_reflectivity[i, :], 'o', label='ground truth')
    plt.semilogy(q, predicted_test_reflectivity[i, :], label='predicted')
    plt.legend()
    plt.xlabel('q [1/A]')
    plt.ylabel('Reflectivity [norm.]')
    plt.show()
```

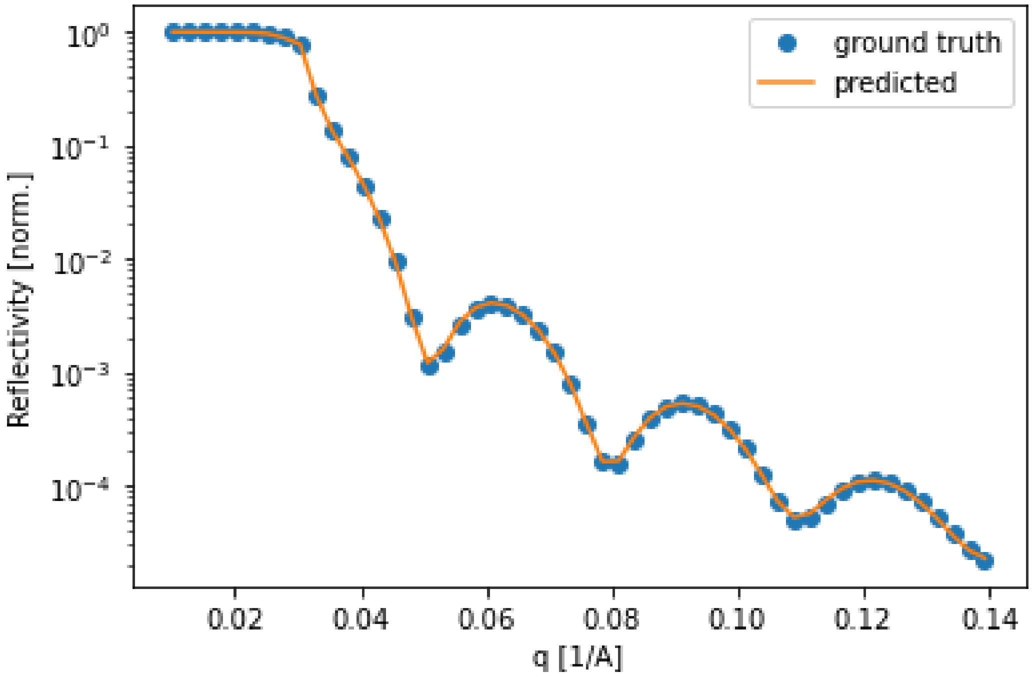

ground truth
predicted
Reflectivity [norm.]
q [1/A]

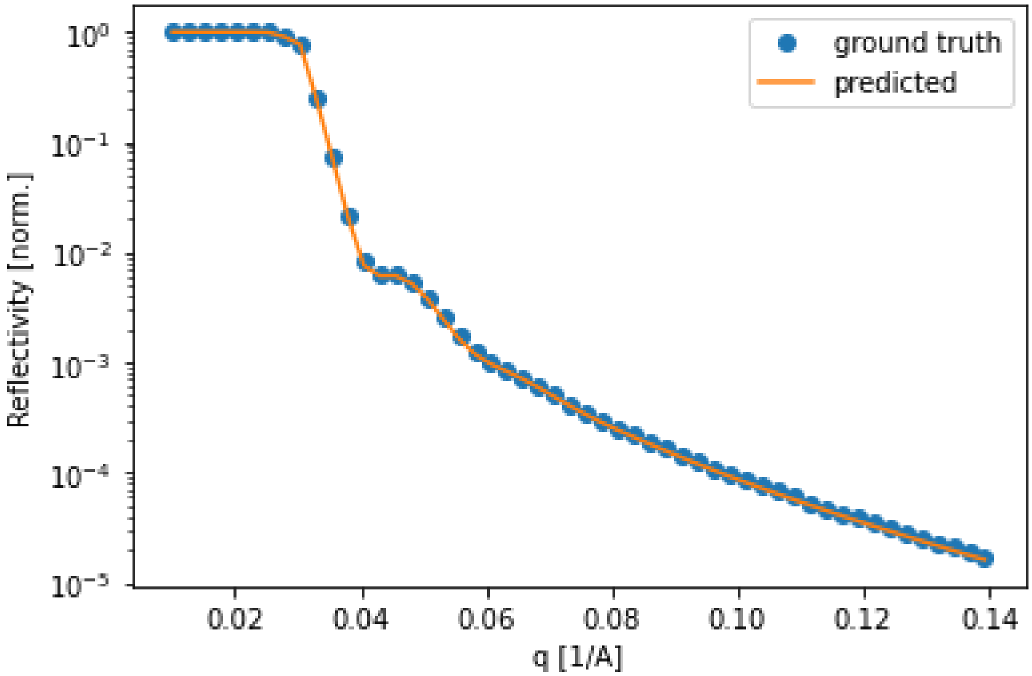

ground truth
predicted
Reflectivity [norm.]
q [1/A]

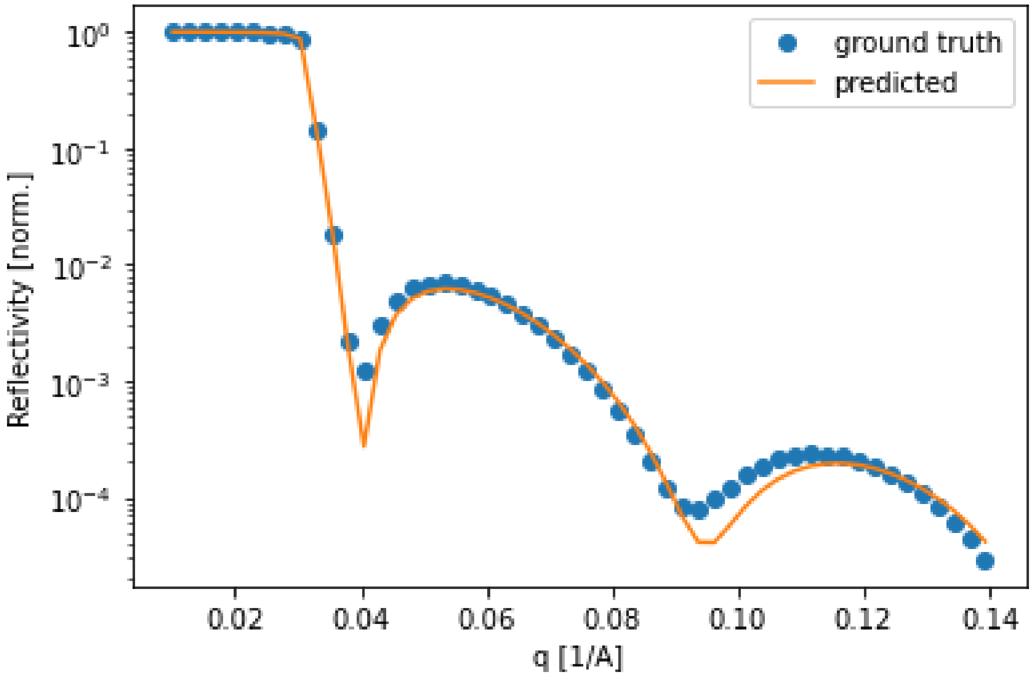

1.4.2 Testing on experimental data

Since the testing on simulated data went so well, we can now try our model on the experimental data mentioned in the beginning. The usage is the same as for the simulated testing data, but this time we turn on `polish` and `optimize_q`.

[35]:
```
experimental_fit_output = curve_fitter.fit_curve(experimental_reflectivity, q,␣
↪polish=True, optimize_q=True)
pred_experimental_reflectivity =␣
↪experimental_fit_output['predicted_reflectivity']
pred_experimental_test_labels = experimental_fit_output['predicted_parameters']
```

[36]:
```
pred_experimental_test_labels.head(5)
```

[36]:
```
   SiOx_thickness  Film_thickness  Si_roughness  SiOx_roughness  \
0              10       19.041166             1             2.5
1              10       20.422161             1             2.5
2              10       20.865399             1             2.5
3              10       19.076324             1             2.5
4              10       20.802391             1             2.5

   Film_roughness               Si_sld              SiOx_sld  Film_sld  \
0        5.586527  20.070100+0.457100j  17.773500+0.404800j  1.990388
1        7.414714  20.070100+0.457100j  17.773500+0.404800j  2.353976
```

```
2        8.033274  20.070100+0.457100j  17.773500+0.404800j  3.598239
3        8.178289  20.070100+0.457100j  17.773500+0.404800j  5.116170
4        6.319250  20.070100+0.457100j  17.773500+0.404800j  5.581930

   ambient_sld
0            0
1            0
2            0
3            0
4            0
```

Since the data is a time series, we can plot the predicted parameters as a function of time. The expected linear growth is observed. Furthermore we see that the roughness increases with thickness (which is expected) and the SLD saturates at a value of about 12 1e-6 1/Å^2, which is a typical value for a DIP thin film.

[37]:
```
plt.plot(pred_experimental_test_labels['Film_thickness'])
plt.xlabel('Time [arbitrary units]')
plt.ylabel('Thickness [A]')
plt.show()

plt.plot(pred_experimental_test_labels['Film_roughness'])
plt.xlabel('Time [arbitrary units]')
plt.ylabel('Roughness [A]')
plt.show()

plt.plot(pred_experimental_test_labels['Film_sld'])
plt.xlabel('Time [arbitrary units]')
plt.ylabel('SLD [1e-6/A^2]')
plt.show()
```

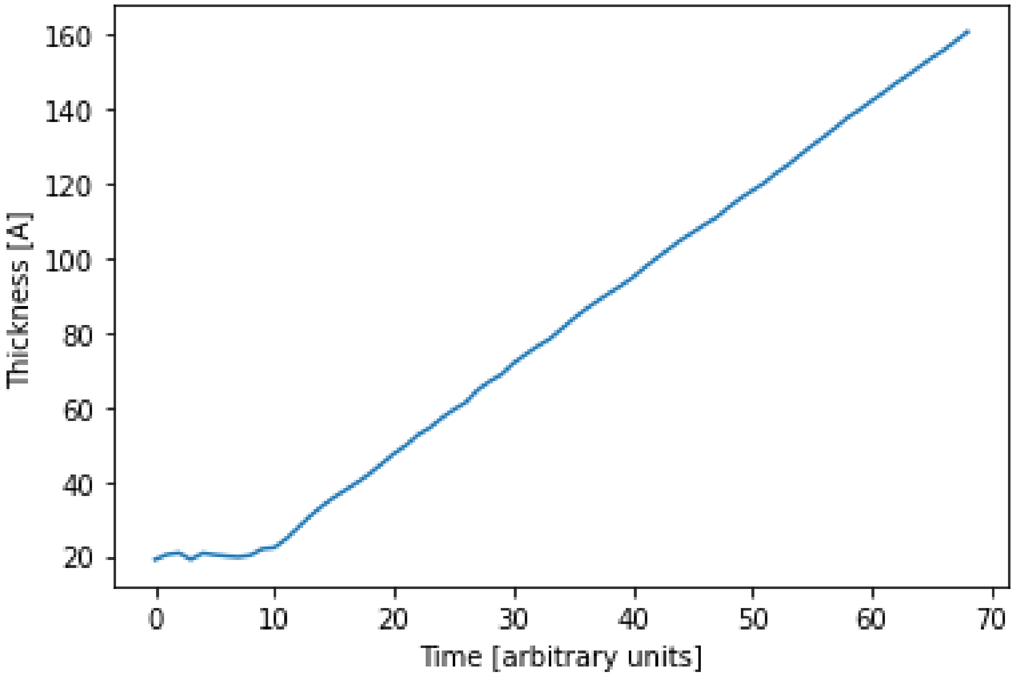
160
140
120
100
80
60
40
20
Thickness [A]
0
10
20
30
40
50
60
70
Time [arbitrary units]

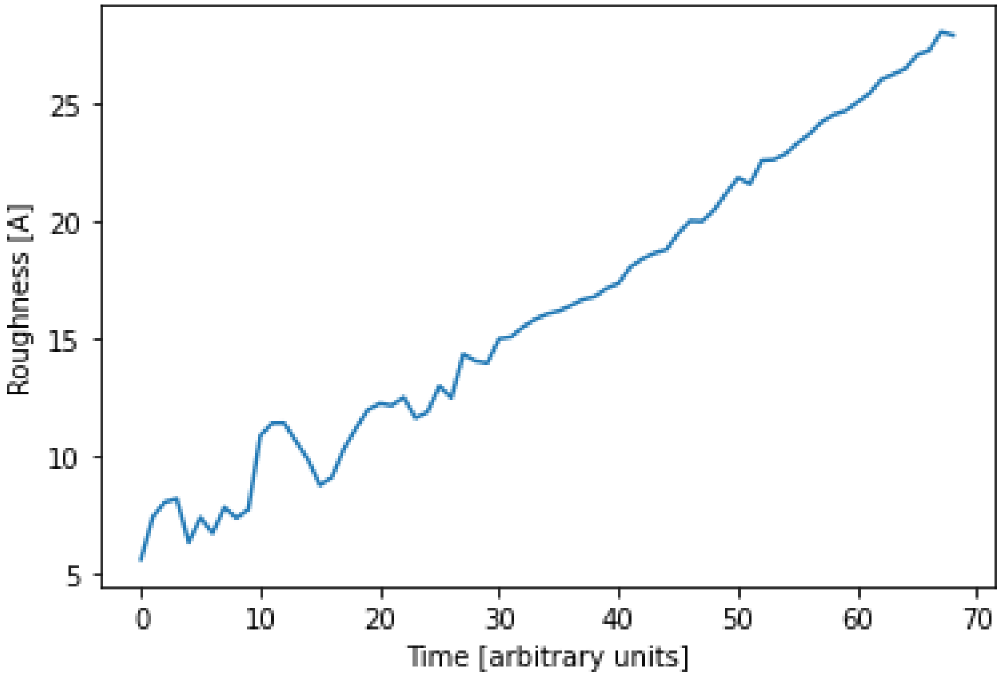
25
20
15
10
5
Roughness [A]
0
10
20
30
40
50
60
70
Time [arbitrary units]

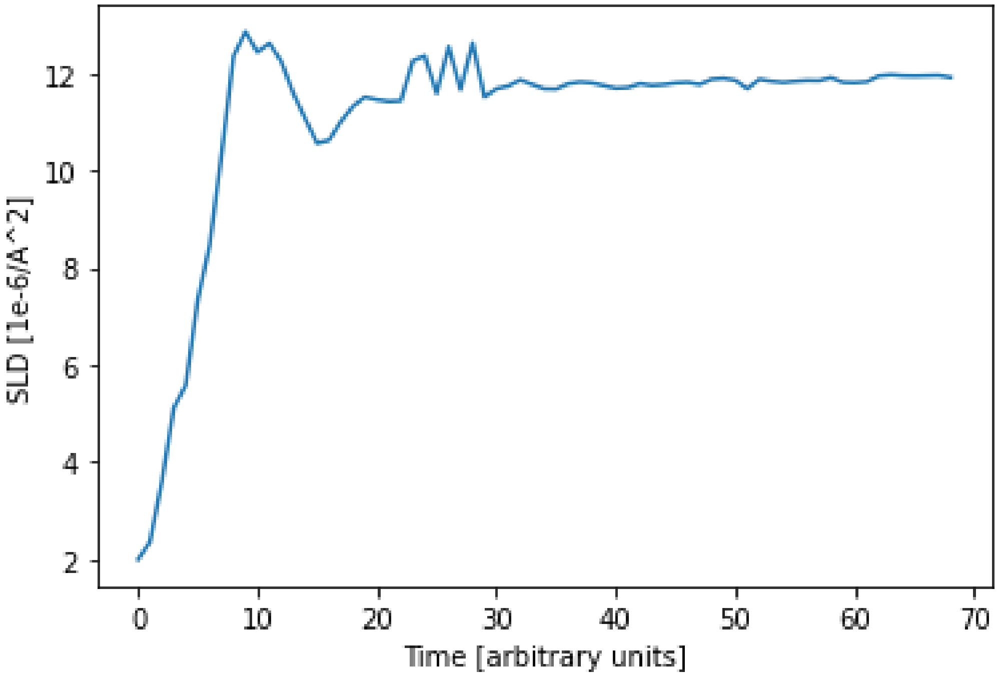

Of course it is important to compare how the simulated curve fits the data. In this example, the curves predicted by the neural network predict the data very well, which inspires confidence in the predicted parameters.

[38]:
```
for i in [15, 30, 50, 68]:
    plt.semilogy(q, experimental_reflectivity[i, :], 'o', label='experiment')
    plt.semilogy(q, pred_experimental_reflectivity[i, :], label='prediction')
    plt.legend()
    plt.xlabel('q [1/A]')
    plt.ylabel('Reflectivity [norm.]')
    plt.show()
```

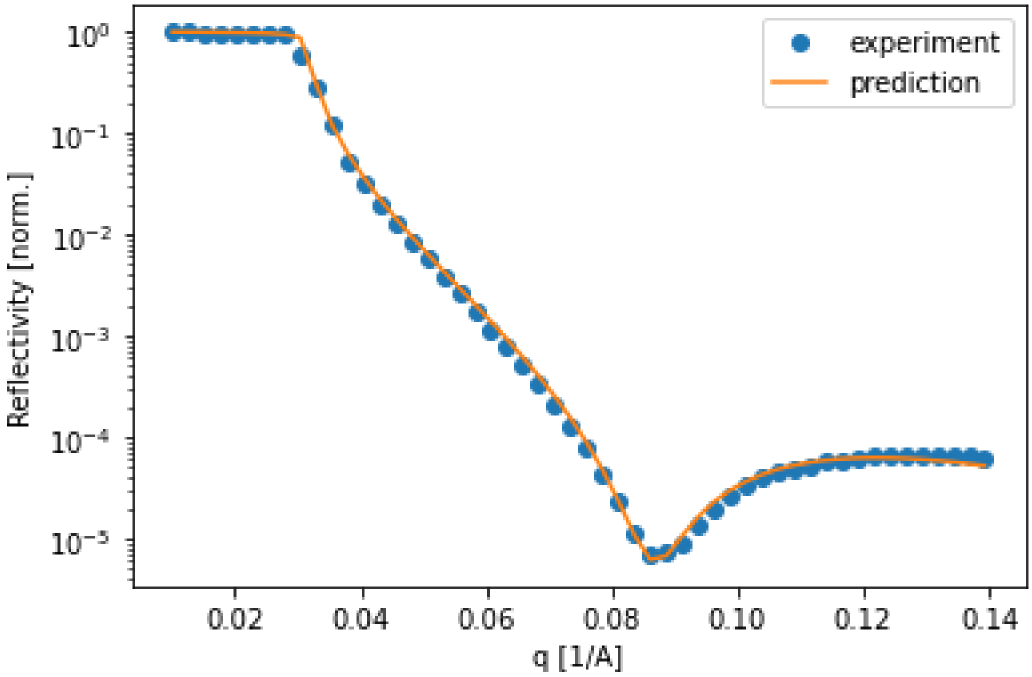
experiment
prediction
Reflectivity [norm.]
q [1/A]
0.02
0.04
0.06
0.08
0.10
0.12
0.14

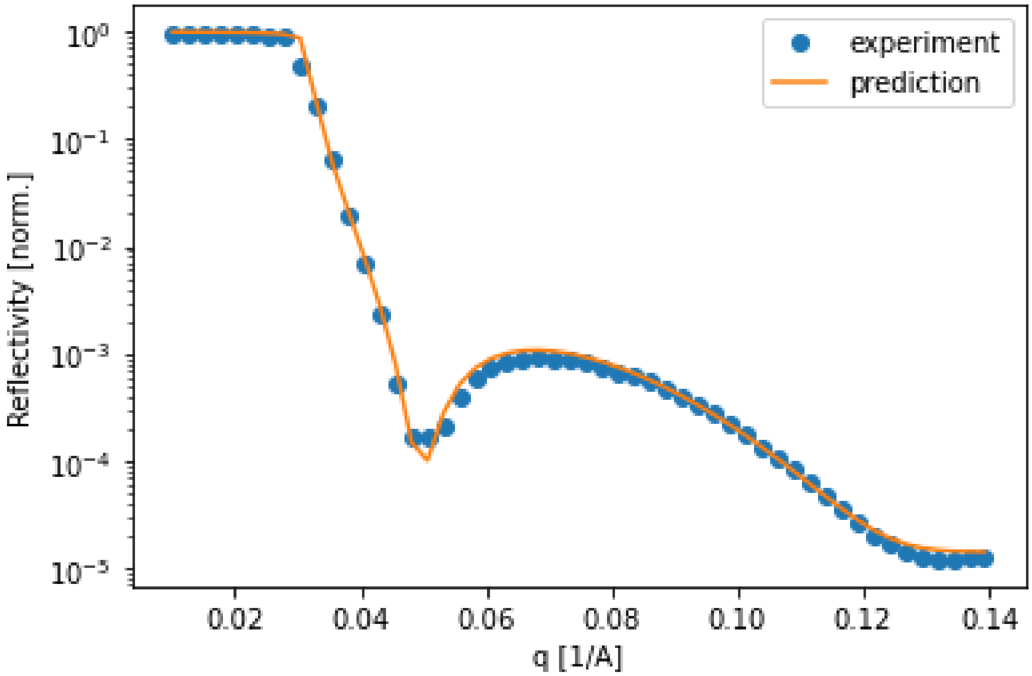
experiment
prediction
Reflectivity [norm.]
q [1/A]
0.02
0.04
0.06
0.08
0.10
0.12
0.14

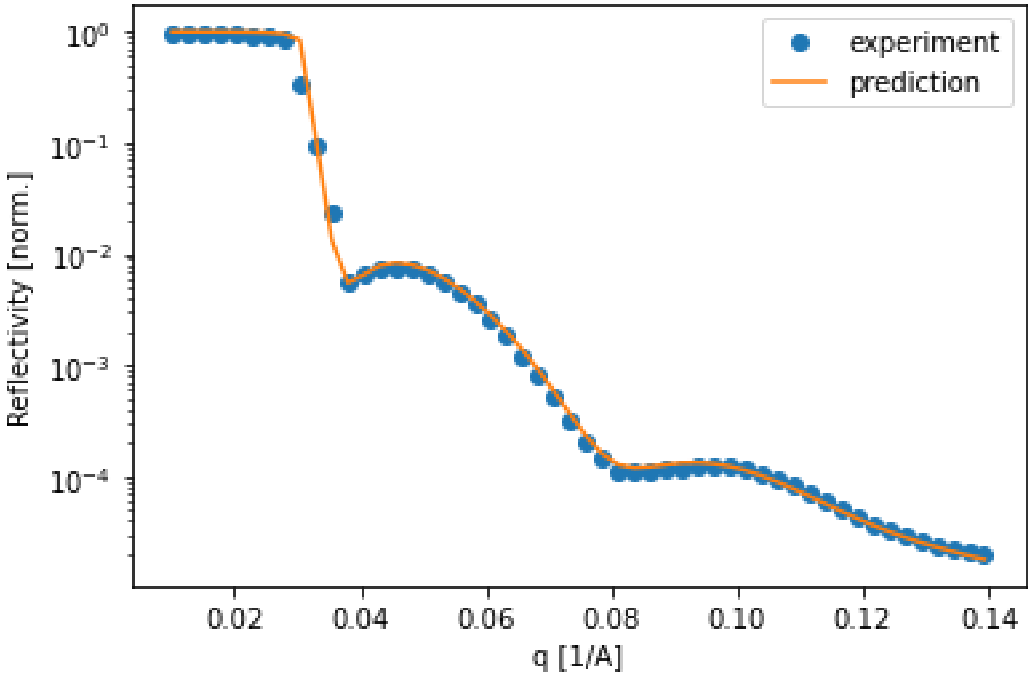

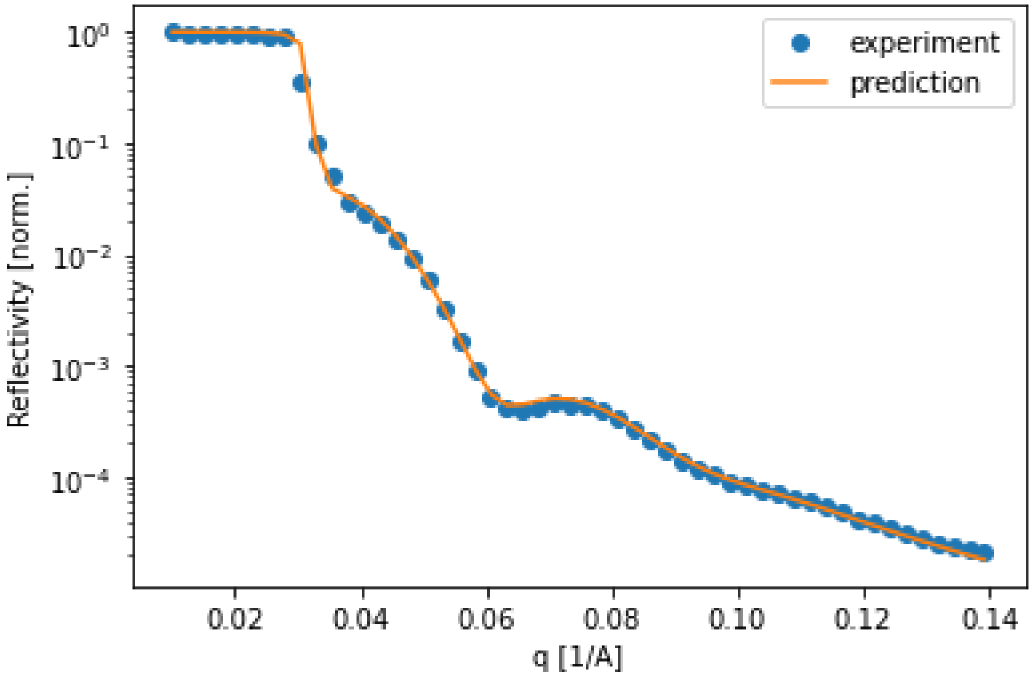

The `TrainedModel` object obtained from `Trainer.train()` can also be used with the `SpecFitter` class to fit data directly from a SPEC file.

This would be done after the `SpecFitter` object is initialized *via* the `set_trained_model()` method.

[39]:
```
from mlreflect.curve_fitter import SpecFitter
```

[40]:
```
spec_fitter = SpecFitter()
spec_fitter.set_trained_model(trained_model)
```

Of course the other parameters of the `SpecFitter` still have to be set (refer to the SpecFitter example).

[ ]:
```
```
